# Supplementary material for: eQTL mapping in fetal-like pancreatic progenitor cells reveals early developmental insights into diabetes risk
Source: Nat Commun. 2023 Oct 30;14:6928. doi: 10.1038/s41467-023-42560-4 (PMC10616100; doi:10.1038/s41467-023-42560-4)
Supplement: Supplementary file 1 — Supplementary Information [file 41467_2023_42560_MOESM1_ESM.pdf]

Supplemental Note 1: Characterization of iPSC-PPC fetal-like transcriptomes using scRNA-seq

Pancreatic progenitor cells (PPCs) are multipotent stem cells that have differentiated beyond the pancreatic foregut and can give rise to all pancreatic cell types (both endocrine and exocrine). PPCs are marked by co-expression of PDX1 and NKX6-1, which we quantified using flow cytometry (Figure 1B, Supplementary Figure 2-3, Supplementary Data 2). To characterize the cellular composition of iPSC-PPCs, we performed scRNA-seq on one iPSC clone (for PPC034) and ten iPSC-PPC samples (Supplementary Data 2). For four of the ten iPSC-PPC samples, we collected both freshly prepared (i.e., not cryopreserved) and cryopreserved cells to examine the effects of cryopreservation on expression quantification. For the remaining six iPSC-PPC samples, the cells were captured only as cryopreserved cells.

We detected eight distinct cell type clusters in iPSC-PPCs (Supplementary Data 2, Supplementary Data 3, Supplementary Data 4). To annotate each cluster, we compared the expression levels of pancreas development marker genes to those in the eight PPC cell types identified in a reference dataset (Supplementary Figure 4-6) [1]. The Veres et al. study [1] performed scRNA-seq over four stages of embryonic stem cell derived-PPC (ESC-PPC) differentiation and included cells from early onset of differentiation (pancreas induction) to more advanced stages (endocrine and exocrine induction), thus providing a valuable benchmark to characterize stem cell-derived PPC cellular heterogeneity. One of the eight clusters in our dataset expressed high levels of *POU5F1*, indicating that this cluster corresponded to the iPSC sample (Supplementary Figure 4-6, Supplementary Data 4). For the iPSC-PPC clusters, we identified: 1) early PPC, which expressed *GATA4*, *GATA6*, and *PDX1*, but not *NKX6-1* and corresponded to *PDX1*$^+$ progenitors in the Veres et al. study; 2) late PPC, which expressed both *PDX1* and *NKX6-1* and corresponded to *NKX6-1*$^+$ progenitors in the Veres et al. study; 3) endocrine cells, which expressed endocrine markers (*PAX6*, *CHGA*) and pancreatic hormones (*INS*, *GCG*, *SST*); and 4) non-endocrine cells, which expressed endothelial markers (*ESM1*, *FLT1*, *PLVAP*) but not mature ductal marker *KRT19* or mature acinar marker *PRSS1*, suggesting that these cells were likely ductal precursors (herein referred to as “early ductal”). We also identified a sub-population within late PPCs that expressed cell division markers (*TOP2A*, *CENPF*, and *AURKB*), indicating that these cells were replicating late PPC and corresponded to replicating *NKX6-1*$^+$ progenitors in the Veres et al. study (labeled as “replicating stage 4” in Supplementary Figure 4). Of note, we identified two cell types from very early pancreas development that were not captured in the Veres et al. dataset, one of which represented mesendoderm (*COL1A1/2*) and the other early definitive endoderm (“early DE”; *AFP*, *APOA2*).

We next examined the extent to which iPSC-PPC cellular heterogeneity was reflected in the flow cytometry analysis. We found that the percentage of late PPCs (including replicating cells) in scRNA-seq was significantly correlated with the percentage of PDX1$^+$/NKX6-1$^+$ cells measured by flow cytometry ($R = 0.857$, $p = 0.00154$, Pearson’s correlation, Supplementary Figure 7A, Supplementary Data 3). We also deconvoluted cell type proportions in bulk RNA-seq samples of iPSC-PPC using cell type-specific markers in scRNA-seq and found that the estimated proportions for late PPC highly corresponded with flow cytometry measurements of PDX1$^+$/NKX6-1$^+$ cells ($R = 0.702$, $p < 2.2\text{x}10^{-16}$, Pearson’s correlation, Supplementary Figure 7B, Supplementary Data 5). These results show that the flow cytometry analysis accurately captured the fraction of late PPCs in the 107 iPSC-PPCs.

We next asked whether cryopreservation affected gene expression profiling in iPSC-PPC. Because gene expression differences between samples are largely driven by cellular heterogeneity, we sought to compare the cell type proportions in scRNA-seq between cryopreserved cells and freshly prepared cells for the four iPSC-PPC samples sequenced with both preparations. We observed that the cellular proportions between both preparations for late PPC, early PPC, mesendoderm, and endocrine were significantly correlated (Supplementary Figure 8, Supplementary Data 3). These results show that cryopreservation did not impact gene expression levels.

Supplemental Note 2: Analysis of iPSC-PPC e_{AS}QTLs

2a. COLOC analysis identifies shared e_{AS}QTLs between the three pancreatic tissues

Similar to the e_gQTL analysis, we performed colocalization between nearby pairs of iPSC-PPC e_iQTLs, adult islet exon eQTLs, and adult whole pancreas splicing eQTLs. Hereafter, we refer to these three different types of eQTLs as e_{AS}QTLs given their functional properties related to alternative splicing [2–4] (Figure 1E). We considered only e_{AS}QTLs that had at least one variant with causal PP ≥ 1%, were outside the MHC region, and associated with genes annotated in GENCODE version 34 [5], therefore retaining 3,959 iPSC-PPC, 4,939 adult islets, and 2,077 adult whole pancreas e_{AS}QTLs. From colocalization, we identified a total of 4,868 pairs of e_{AS}QTLs that displayed high evidence of colocalization with PP.H4 ≥ 80% (Supplementary Data 9). We also observed that e_{AS}QTLs for the same gene may colocalize with each other indicating that a single causal variant may impact multiple splicing processes for a gene.

We next identified tissue-unique singleton and combinatorial e_{AS}QTLs. Using the same approach in the e_gQTL analysis, we identified 631 iPSC-PPC, 1,522 adult islets, and 431 adult whole pancreas singleton e_{AS}QTLs (i.e., e_{AS}QTLs that neither colocalized nor were in LD ($r^2 < 0.2$ within 500 Kb or outside of 500 Kb if LD not available) with nearby e_{AS}QTLs, indicating that their underlying causal variants affect alternative splicing of a single transcript specifically during early pancreas development or in one of the adult pancreatic tissues (Supplementary Figure 11A, Supplementary Data 9, Supplementary Data 10). To identify tissue-unique combinatorial e_{AS}QTLs, we created a network using the 4,868 pairwise colocalizations and then filtered modules that failed specific module and LD criteria (see Methods). We identified 980 e_{AS}QTL modules in total, averaging ~3 e_{AS}QTLs per module (range: 2-13) (Supplementary Data 10, Supplementary Data 11). 344 (35.1% of 980) e_{AS}QTL modules were tissue-unique, of which 124 were fetal-like iPSC-PPC-unique, 203 adult islet-unique, and 17 adult whole pancreas-unique, and comprised 266, 452, 37 e_{AS}QTLs, respectively (Supplementary Figure 11B). The remaining 636 (64.9% of 980) e_{AS}QTL modules were shared between multiple pancreatic tissues, 225 of which were shared between only the two adult pancreatic tissues ("adult-shared"), 139 shared between iPSC-PPC and adult islets ("fetal-islet"), 58 shared between only iPSC-PPC and adult whole pancreas ("fetal-whole-pancreas"), and 214 shared between all three pancreatic tissues ("fetal-adult") (Supplementary Figure 11B). Together, the 411 (139 + 58 + 214) modules shared between iPSC-PPC and an adult pancreatic tissue comprised 802 iPSC-PPC, 561 adult islets, and 318 adult whole pancreas e_{AS}QTLs (Supplementary Data 10, Supplementary Data 11).

Altogether, from this analysis, we identified 897 (22.7% of 3,959) iPSC-PPC-unique e_{AS}QTLs, of which 631 (70.3%) functioned as singletons and 266 (29.7%) in modules, while 802 (20.3% of 3,959) were shared with at least one adult pancreatic tissue (Supplementary Data 10, Supplementary Data 11). The remaining 2,260 (57.1% of 3,959) iPSC-PPC e_{AS}QTLs failed either module or LD criteria (see Methods). In adult whole pancreas, we observed much fewer tissue-unique

e_{AS}QTL modules compared to e_gQTLs because each gene only had one significant sQTL and therefore unlikely to colocalize with one another. On the other hand, in iPSC-PPCs and adult islets, genetic variants affect the expression of multiple isoforms or exons corresponding to the same gene in the same tissue; and hence, compared with the e_gQTLs, a greater fraction of the iPSC-PPC-unique and the adult islet-unique e_{AS}QTLs were combinatorial. Similar to the e_gQTLs, the vast majority of tissue-unique regulatory variants were singletons, potentially due to large differences in alternative splicing between fetal and adult pancreatic tissues, which is known to exist for other tissue-types [6–8]. Additionally, there may be other distinct properties e_iQTLs, exon eQTLs, and splicing eQTLs that we did not account for.

2b. Characterization of fetal-adult-shared e_{AS}QTL modules in iPSC-PPC

We next determined the fraction of shared genetic loci associated with alternative splicing of the same or different genes between fetal-like iPSC-PPC and the two adult pancreatic tissues. Similar to the e_gQTL analysis, we focused on the 411 e_{AS}QTL modules with both iPSC-PPC and adult e_{AS}QTLs (“fetal-adult”, “fetal-islet”, “fetal-whole-pancreas”) and compared the genes associated with each module. We identified: A) 149 modules that were associated with the same gene between fetal-like iPSC-PPC and only one of the two adult pancreatic tissues (1 gene per module); B) 85 modules associated with the same gene in all three tissues (1 gene per module); C) 93 modules associated with 2-5 genes, of which some genes were shared but at least one gene was different between iPSC-PPC and at least one adult tissue; D) 57 modules associated with different genes between iPSC-PPC and only one of the two adult tissues (2-5 genes per module); E) the remaining 27 modules associated with different genes between fetal-like and both the two adult tissues (range: 2-5 genes per module) (i.e., there is no overlap of genes between the two developmental stages; Supplementary Figure 11C, Supplementary Data 11). 43.1% (93 + 57 + 27 = 177 / 411; categories C-E) of the fetal-adult-shared modules displayed functional plasticity, in which the underlying regulatory variants were associated with splicing events for multiple different genes. These modules comprised 386 iPSC-PPC, 361 adult islets, and 184 adult whole pancreas e_{AS}QTLs (Supplementary Data 10, Supplementary Data 11).

Supplementary Figure 1: Subject and sample characteristics of iPSC-PPC cohort

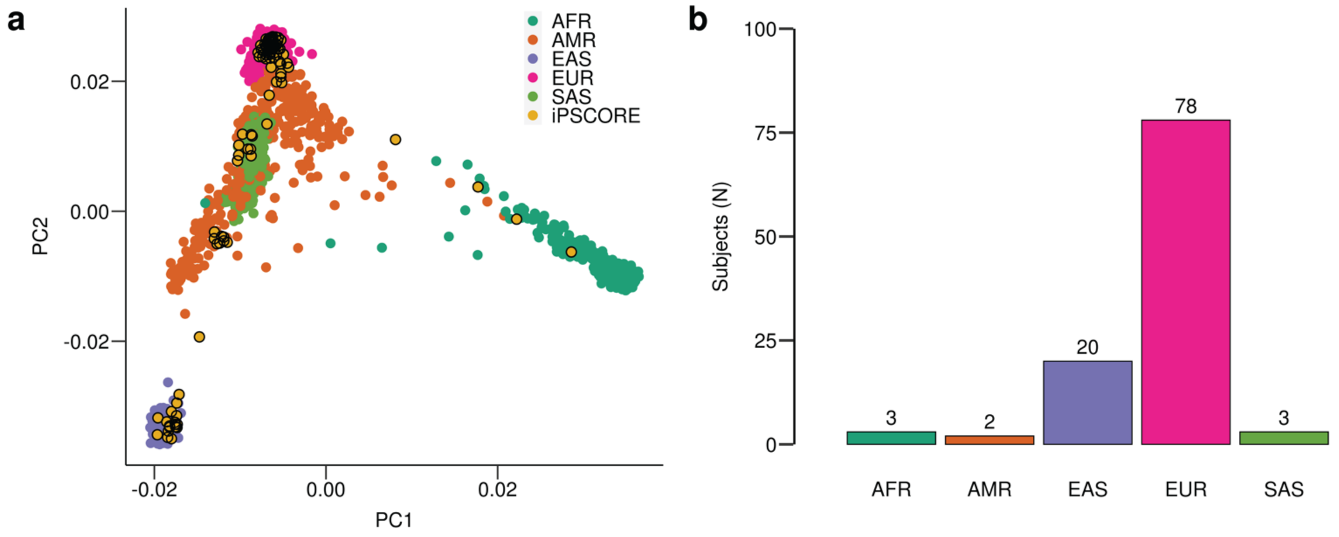

(**a**) Scatter plot showing the first two genotype principal components of the 106 iPSCORE subjects in relation to the 2,504 subjects in the 1000 Genomes Project (1KGP) Phase 3 dataset. Color indicates the five 1KGP superpopulations (AFR, AMR, EAS, EUR, and SAS). Black bordered yellow circles correspond to the 106 iPSCORE individuals in this study (Supplementary Data 1). (**b**) Bar plot showing the distribution of the 106 iPSCORE subjects across different 1KGP superpopulations. Subjects were assigned to the most similar 1KGP population in a previous study using linear discriminant analysis [9].

Supplementary Figure 2: Measurement of PDX1$^+$ and NKX6-1$^+$ by flow cytometry for 107 iPSC-PPC samples

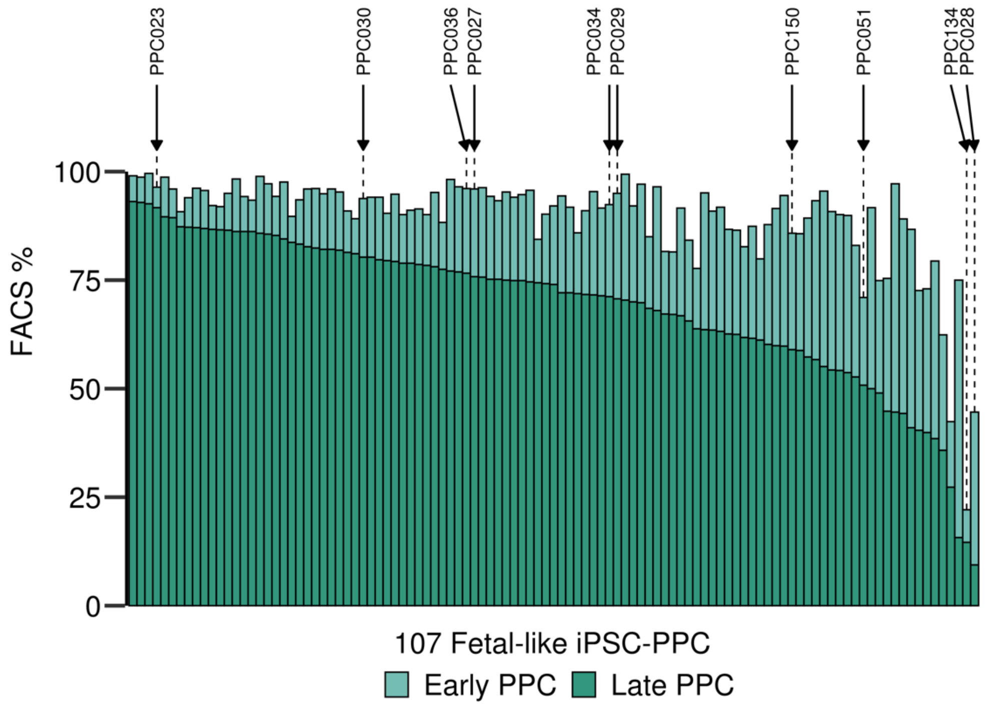

Bar plot showing the percentage of late PPC and early PPC cells detected by FACs for all of the 107 differentiations (Supplementary Data 2). The ten highlight samples by arrows indicate samples used for scRNA-seq examination. We show that the samples selected for scRNA-seq varied in late PPC percentage. The two iPSC-PPC samples derived from the same iPSC line (PPC029 and PPC036) had similar percentages of late PPCs (70.7% and 76.6%, respectively).

Supplementary Figure 3: Flow cytometry results for the ten iPSC-PPC samples used in single-cell analysis

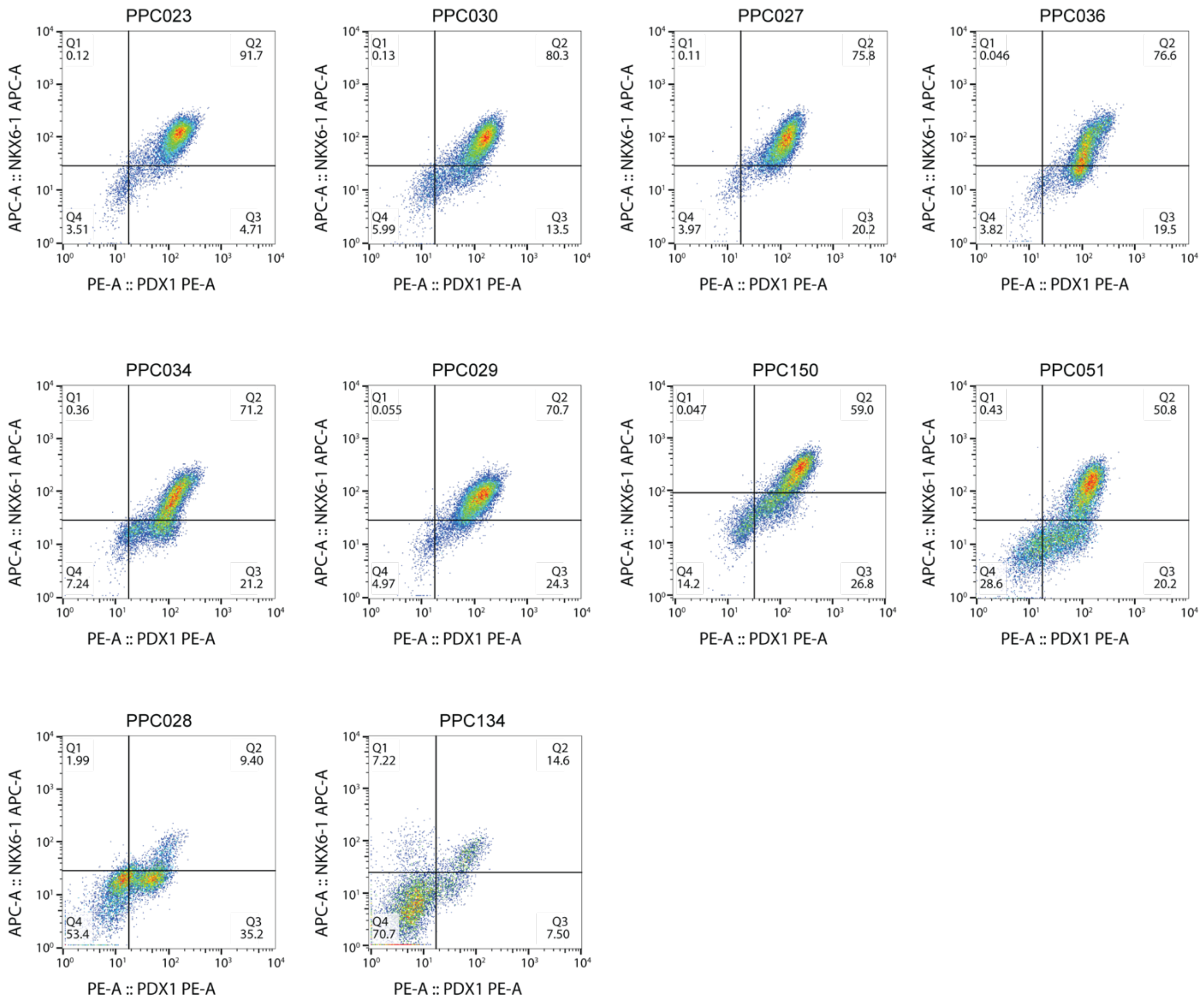

Flow cytometry analysis at D15 of the ten iPSC-PPC samples that underwent scRNA-seq (Supplementary Figure 2). The percentage of cells stained for PDX1 (X-axis) and NKX6-1 (Y-axis) were measured. Differentiations PPC029 and PPC036 were from the same iPSC line and showed similar percentages of double-positive cells. Measurements for all 107 iPSC-PPC differentiations are provided in Supplementary Data 2 and shown in Supplementary Figure 2.

Supplementary Figure 4: Single cell characterization of fetal-like iPSC-PPC

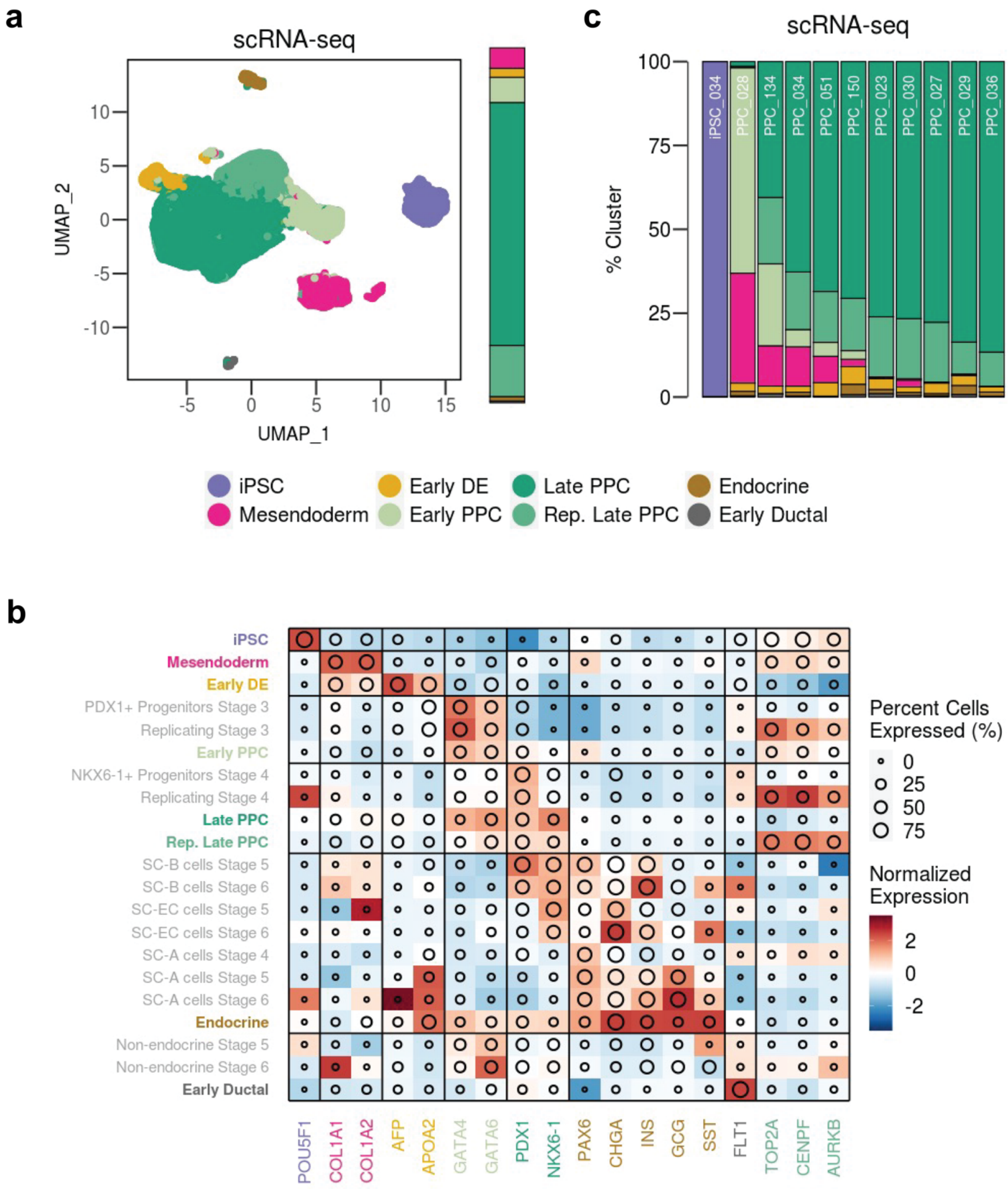

We characterized the cellular composition of iPSC-PPC using scRNA-seq of one iPSC (for PPC034 differentiation) and ten iPSC-PPC samples with variable percentages of double-positive cells (range: 9.4% - 91.7%) (Supplementary Figure 2, Supplementary Figure 3). We identified eight distinct cell populations, corresponding to iPSC (*POU5F1*), mesendoderm (*COL1A1/2*), early definitive endoderm (early DE; *AFP*, *APOA2*), early PPC (*GATA, GATA6, PDX1*), late PPC (*PDX1* and *NKX6-1*), replicating late PPC (*PDX1, NKX6-1, TOP2A, CENPF, AURKB*), endocrine (*PAX6, CHGA, INS, GCG, SST*), and early ductal (*FLT1*) (Supplementary Figure 4C, Supplementary Data 3). We observed highly similar gene expression

profiles between the cell types identified in iPSC-PPC and those identified in an ESC-derived PPC (ESC-PPC) reference dataset [1].

(**a**) UMAP plot of scRNA-seq data from 84,225 single cells from one iPSC and ten iPSC-PPC samples. Each point represents a single cell color-coded by its assigned cluster. To the right of the UMAP plot, we show relative proportion of cells associated with each cell type (iPSC cells excluded). We show that the vast majority of cells in iPSC-PPCs were late PPCs. (**b**) Heatmap comparing the Z-normalized expression of known marker genes between iPSC-PPC and cells from the reference ESC-PPC study [1]. Color intensity indicates the mean Z-normalized expression across all cell types, and the diameter indicates the percentage of cells expressing the markers above the threshold of 1% of the maximum expression value. Clusters labeled in color correspond to the iPSC-PPC clusters. Clusters labeled in grey correspond to ESC-PPC clusters [1]. (**c**) Stacked bar plot showing the relative proportion of cells from each sample assigned to each cluster in scRNA-seq. Color-coding corresponds to the clusters in panel **a**. Samples with the least number of late PPC cells correspond to those with weaker differentiation efficiency based on FACS, and contain more cells of primitive state compared to the other samples.

Supplementary Figure 5: Clustering of iPSC-PPC scRNA-seq at two additional resolutions

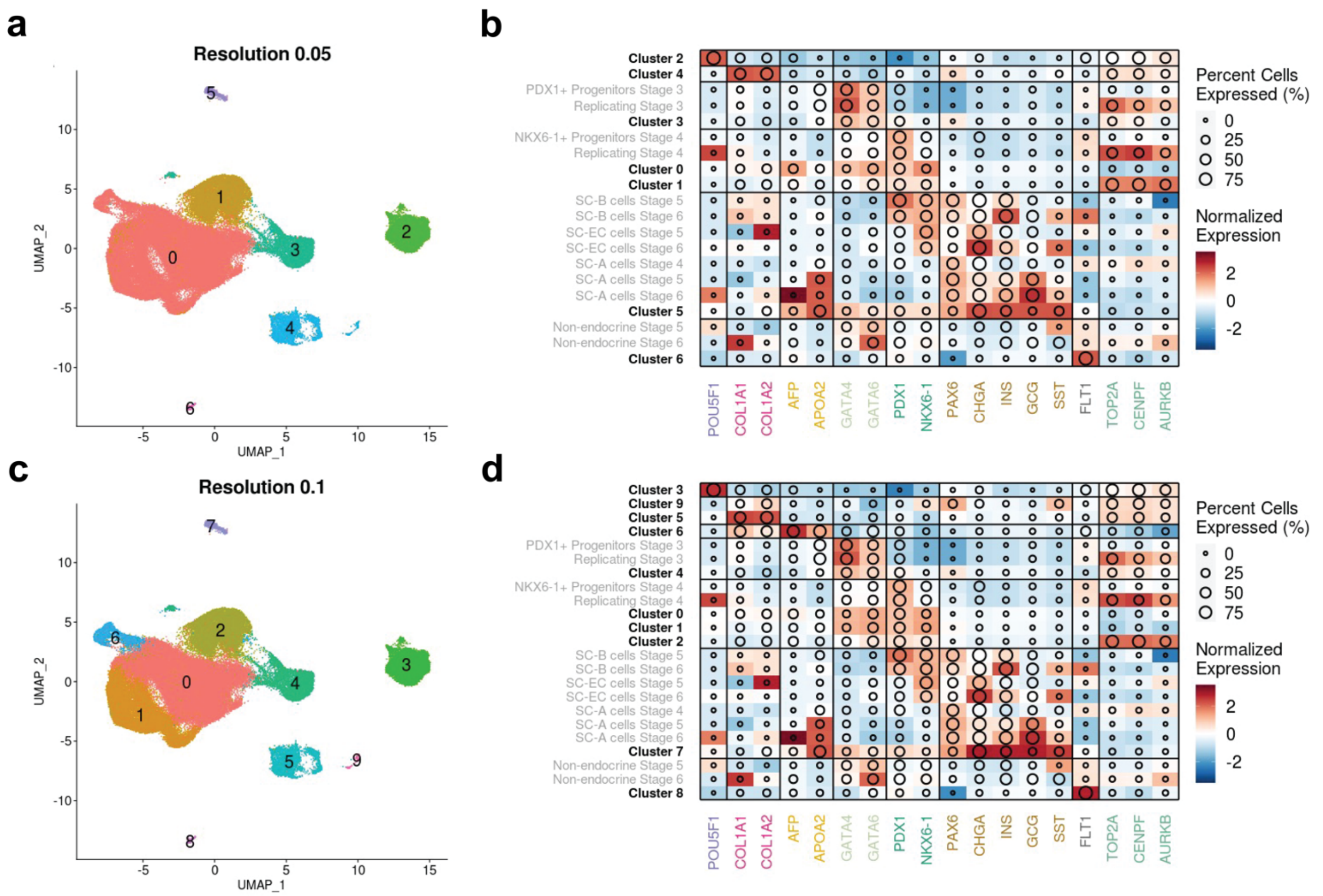

As described in the Methods, we performed clustering at three different resolutions: 0.05, 0.08 (shown in Supplementary Figure 4A), and 0.1, to annotate cell populations in the fetal-like iPSC-PPCs. Compared with resolution 0.08, resolution 0.05 (panel **a, b**) collapsed both early DE and late PPC as cluster 0, while resolution 0.1 (panel **c**) further divided late PPCs into two subclusters (cluster 0 and cluster 1). For resolution 0.1, we examined the expression profiles of the two subclusters in late PPCs and found that cluster 1 expressed similar levels of *PDX1* and *NKX6-1* as cluster 0 (panel **d**). Therefore, we used resolution 0.08 for downstream analyses (Supplementary Figure 4A). Color intensity indicates the mean Z-normalized expression across all cell types, and the diameter indicates the percentage of cells expressing the markers above the threshold of 1% of the maximum expression value. Cluster labels in black correspond to the iPSC-PPC clusters in the UMAP plots on the left. Cluster labels in grey correspond to ESC-PPC clusters [1].

Supplementary Figure 6: Expression of 18 marker genes in each of the eight scRNA-seq clusters

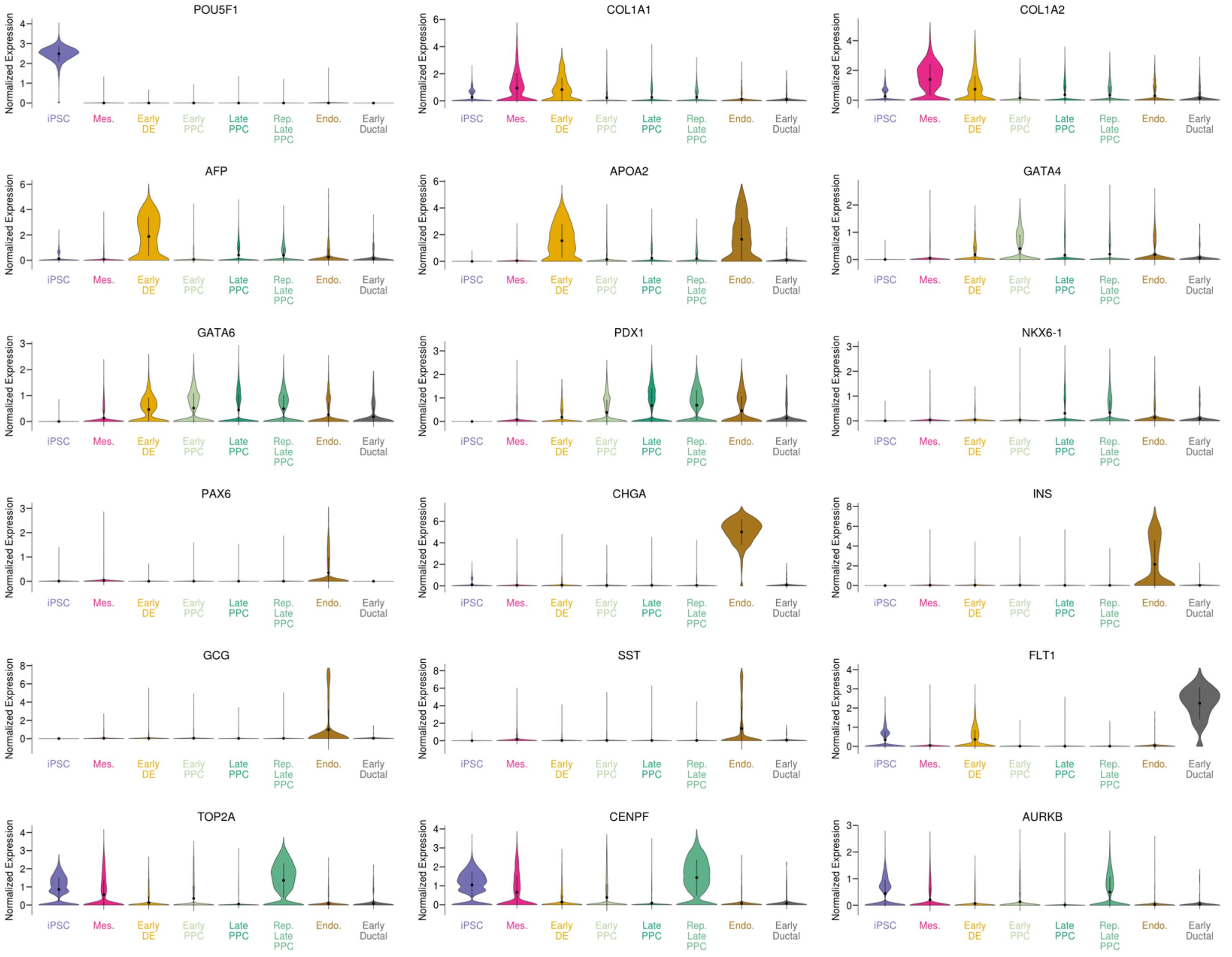

Violin plots showing the distribution of normalized expression for marker genes described in Supplementary Figure 4B for each iPSC-PPC scRNA-seq cluster in Supplementary Figure 4A. Center points in each violin represents the mean expression value. Error bars represent the standard deviation of expression across the cells.

Supplementary Figure 7: Correlation between flow cytometry and cell type proportions

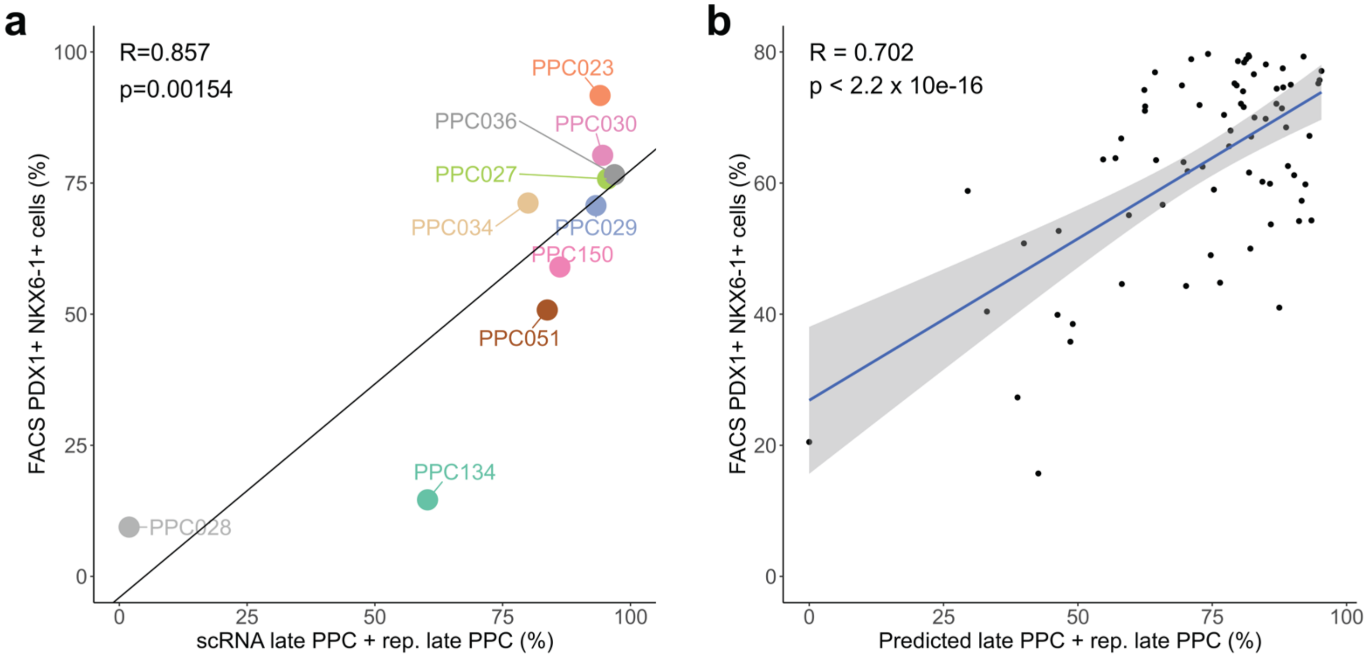

(**a**) To determine the correspondence between flow cytometry and scRNA-seq, we compared the percentage of double-positive cells in each iPSC-PPC samples as measured by flow cytometry (Y-axis; Supplementary Data 2, Supplementary Figure 2, Supplementary Figure 3) to the percentage of cells annotated as late PPC or replicating late PPC in scRNA-seq (X-axis; Supplementary Data 3, Supplementary Figure 4C). We found that independent measurements from flow cytometry and scRNA-seq were positively correlated (R = 0.857, p = 0.00154, spearman correlation). (**b**) We next examined the correspondence between the percentage of late PPCs from FACs (PDX1+/NKX6-1+) (Y-axis; Supplementary Data 2, Supplementary Figure 2, Supplementary Figure 3) and the estimated percentage of late PPCs (including replicating late PPCs) in bulk RNA-seq using CIBERSORTx [10] (X-axis; Supplementary Data 5). As expected, we observed a strong correlation between the two measurements ($R = 0.702$, $p < 2.2 \times 10^{-16}$), indicating that the cellular heterogeneity captured by flow cytometry is represented in the iPSC-PPC bulk expression profiles.

Supplementary Figure 8: Fresh and cryopreserved cells in scRNA-seq

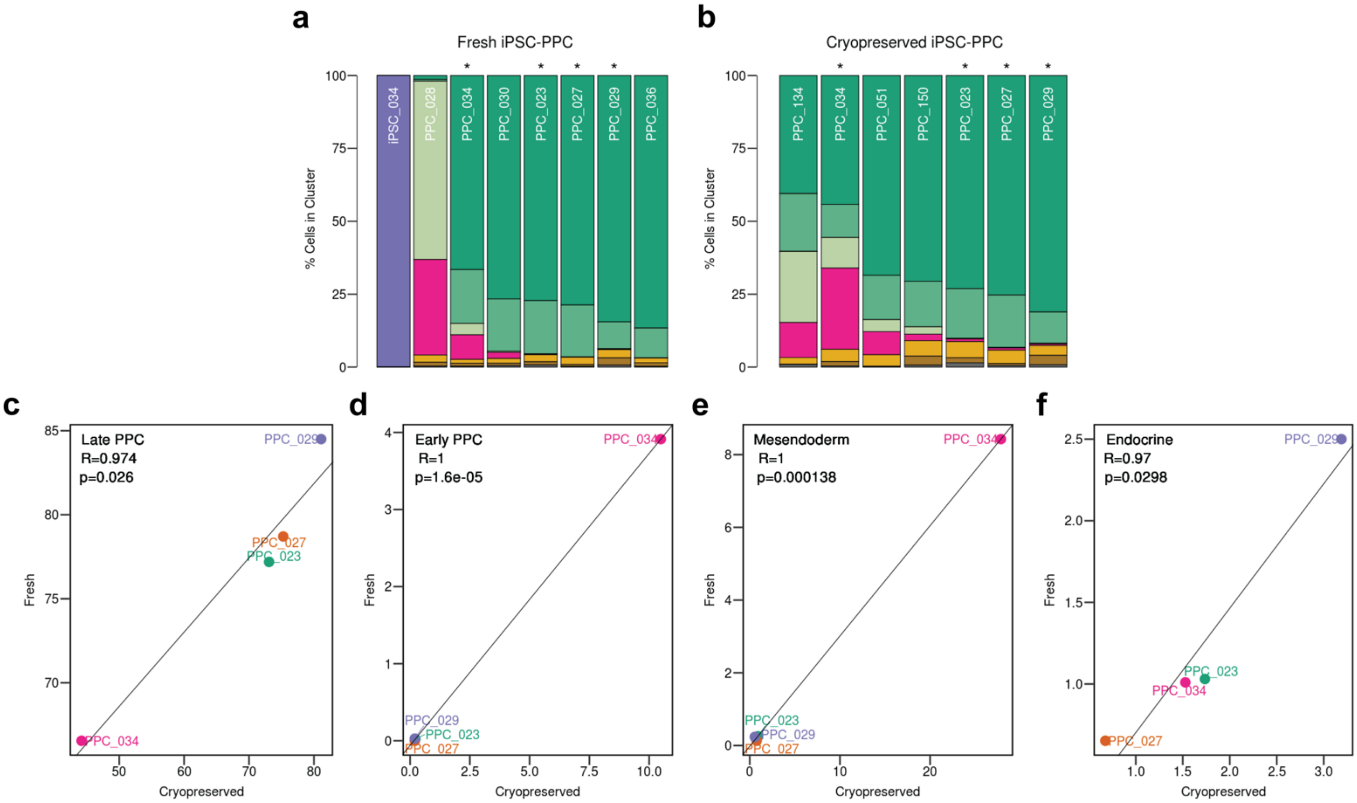

To determine if cryopreservation affects gene expression in iPSC-PPC, we asked whether the cell type proportions differed between matched fresh and cryopreserved samples for four iPSC-PPCs (PPC034, PPC023, PPC027, and PPC029; indicated by an asterisk in panel **a** and **b**). We compared the relative proportion of cells in early PPC, late PPC, mesendoderm, and endocrine clusters, and observed that fresh and cryopreserved samples were highly correlated. These results suggest that cell cryopreservation does not affect relative gene expression between samples, and therefore, can be used to characterize the cellular heterogeneity of iPSC-PPC. The Supplementary Figure hows: (**a**) Stacked bar plots showing the percentage of cells in each of the eight freshly prepared samples (one iPSC and seven iPSC-PPC) according to their cell type using the same color coding as Supplementary Figure 4A. Asterisks indicate the four iPSC-PPC samples with matched cryopreserved preparations. (**b**) Stacked bar plots showing the percentage of cells in each of the seven cryopreserved samples (all iPSC-PPC) according to their cell type using the same color coding as Supplementary Figure 4A. (**c-f**) For the four iPSC-PPC samples with matched fresh and cryopreserved samples, we show the association between each preparation by comparing the percentage of cells for early PPC (**c**), late PPC (**d**), mesendoderm (**e**), and endocrine (**f**).

Supplementary Figure 9: iPSC-PPCs represent a fetal-like state of the pancreas

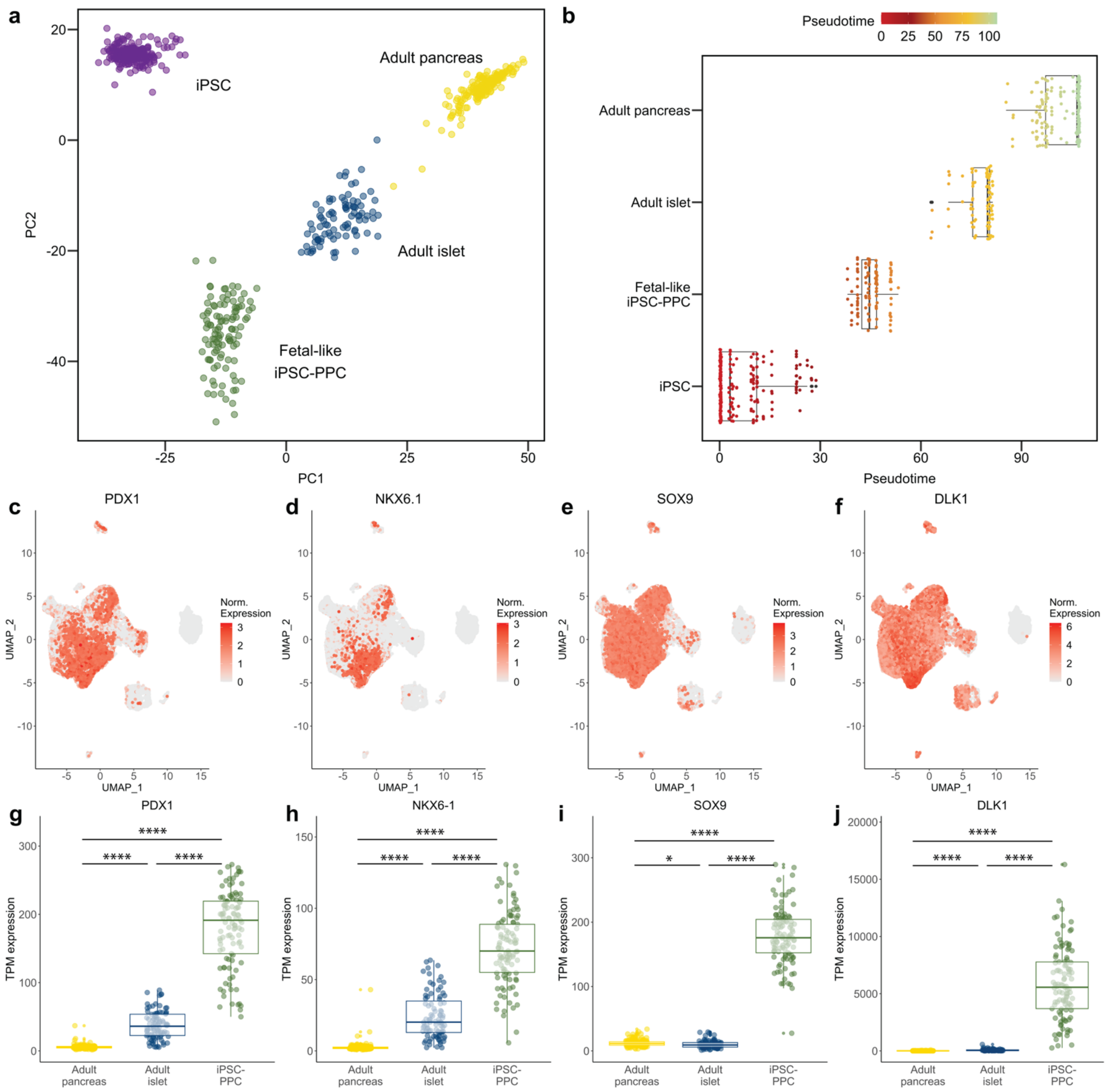

(**a**) Scatter plot showing the PCA distributions of the bulk transcriptomes of 213 iPSCs [11], 107 fetal-like iPSC-PPCs, 87 adult islets [12], and 176 adult whole pancreas [13] using the top most variable genes across all samples in bulk RNA-seq (Supplementary Data 6). (**b**) Box plot showing the pseudotime for each of the four tissues (213 iPSCs, 107 fetal-like iPSC-PPCs, 87 adult islets, and 176 adult whole pancreas) presented as +/- 1.5 interquartile range with the median line denoted at the center. Pseudotime was estimated using Monocle [14] where time was rooted at 0 in iPSCs. (**c-f)** UMAP plots showing the expression of fetal pancreatic genes [15–21] (*PDX1*, *NKX6-1*, *SOX9*, and *DLK1*) in early and late PPCs in iPSC-PPC (cell types labeled in Supplementary Figure 4A). (**g-j)** Box plots showing the expression of the same fetal pancreatic genes in panels **c-f** in bulk RNA-seq of 107 iPSC-PPCs (green), 87 adult islets (blue), and 176 adult whole pancreas (yellow). Paired

student T-test was performed to evaluate the significance of expression differences between tissues. * = $p < 0.05$, ** = $p < 0.01$, *** = $p < 0.001$, **** = $p < 0.0001$. Box plots indicate median interquartile range (IQR), and 1.5 x IQR. These results confirm that iPSC-PPCs represent an early developmental time point compared to adult human islets and whole pancreas.

Supplementary Figure 10: Functional enrichment of overlapping e_gQTLs and e_iQTLs

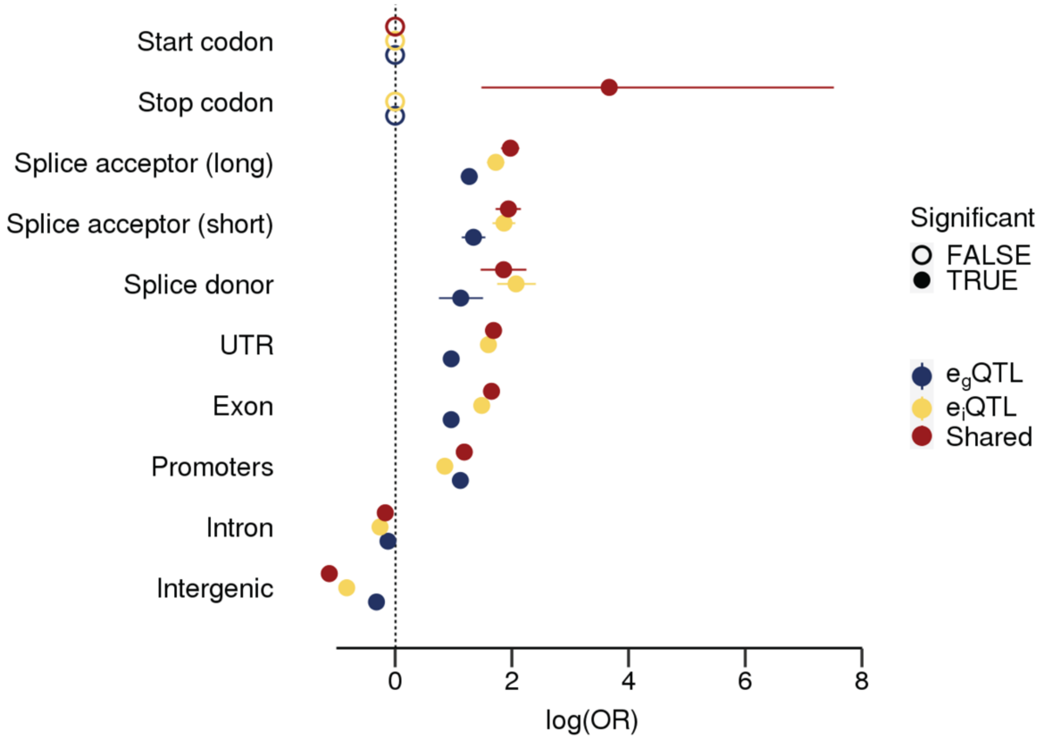

We divided the eQTL signals into three groups: 1) e_gQTLs that did not colocalize with e_iQTLs, 2) e_iQTLs that did not colocalize with e_gQTLs, and 3) eQTLs that had colocalized e_gQTLs and e_iQTLs ("shared", PP.H4 ≥ 80%). For non-colocalized e_gQTL and e_iQTL signals, we obtained all SNPs with causal PP ≥ 5% (Supplementary Data 9). For colocalized eQTLs, we used the predicted causal SNPs underlying both associations (output from colocalization). We intersected the SNP overlap with genomic regions (Y-axis) and performed a two-sided Fisher's Exact Test to calculate the enrichment of each eQTL group against a null background set of 20,000 variants. We found that overlapping eQTL signals were enriched in both regulatory and splice sites while non-overlapping e_gQTL and e_iQTLs were more enriched in their respective regions against each other. For example, e_gQTLs displayed a stronger enrichment for promoter regions compared to e_iQTLs while e_iQTLs were more enriched in splice sites compared to e_gQTLs, consistent with Figure 1E and other studies [2–4]. P-values were Benjamini-Hochberg-corrected and considered significant if the corrected p-values < 0.05. Non-significant results were set to log(odds ratio) = 0. Error bars represent 95% confidence intervals for the odds ratios.

Supplementary Figure 11: Singleton and combinatorial e_{AS}QTLs

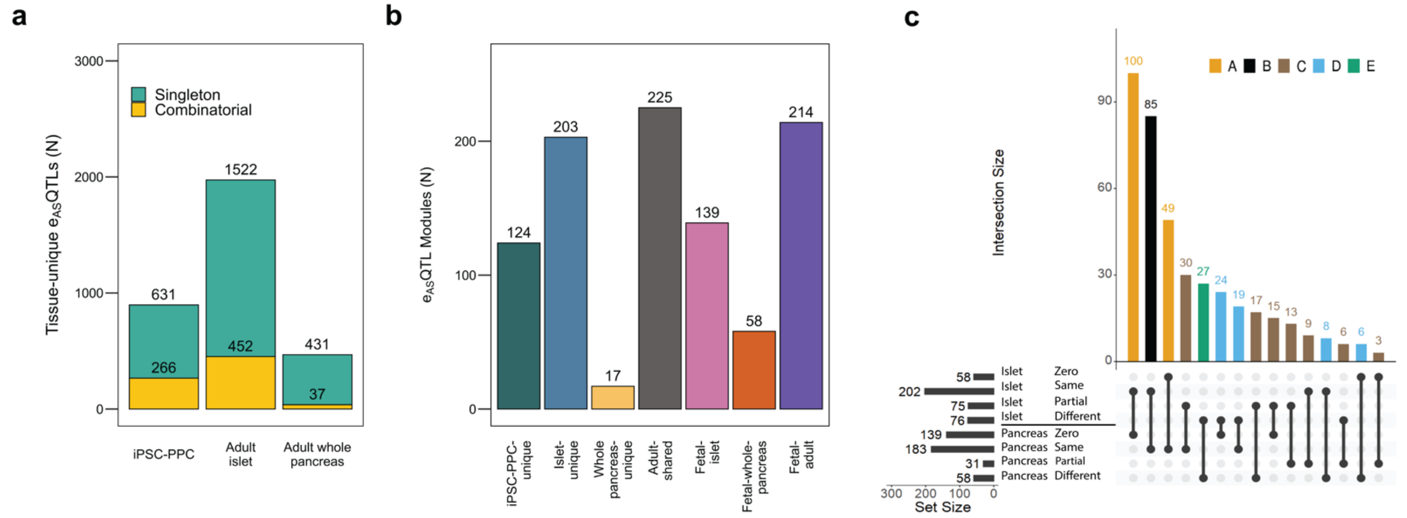

(**a**) Bar plot showing the number of e_{AS}QTLs that were classified as tissue-unique singletons (green) or in combinatorial (yellow) associations in each of the three pancreatic tissues. (**b**) Bar plot showing the number of e_{AS}QTL modules identified for each annotation. See Results and Methods for the description of each module category. (**c**) Number of e_{AS}QTL modules based on eGene overlap between iPSC-PPC and the two adult pancreatic tissues (Supplementary Data 11). "Zero" indicates that the module does not contain an e_{AS}QTL in the respective adult tissue. "Same" indicates that the module contains only e_{AS}QTLs corresponding to the same eGenes in iPSC-PPC and the adult tissue. "Partial" indicates that the module contains e_{AS}QTLs corresponding to partially overlapping eGenes between iPSC-PPC and the adult tissue. "Different" indicates that the module contains only e_{AS}QTLs corresponding to different eGenes between iPSC-PPC and the adult tissue.

Supplementary Figure 12: Definition of a GWAS locus

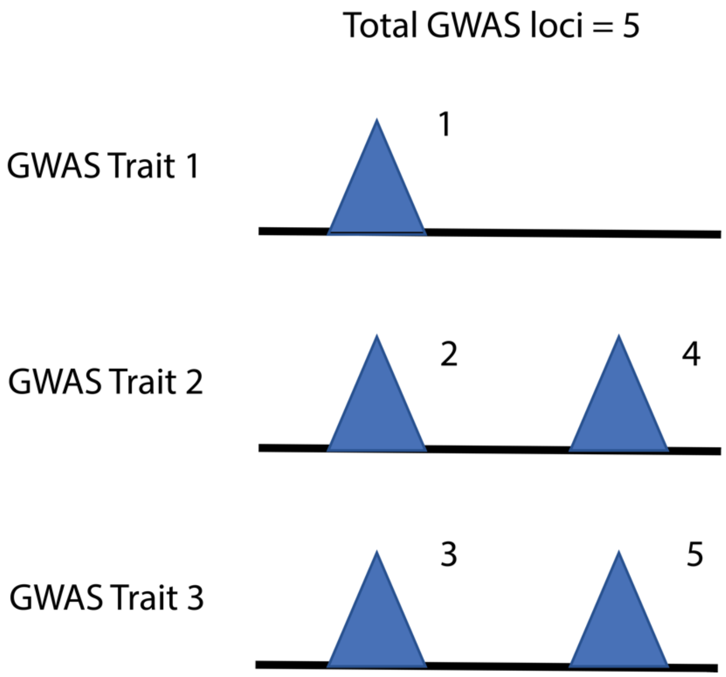

Given that some traits are highly correlated with one another, we observed some eQTLs that colocalized with GWAS variants associated with more than one trait. We considered each combination of colocalized eQTL-GWAS trait variants as a separate GWAS locus. The example in the Supplementary Figure hows two eQTL signals that overlap with either three or two traits, which we count as five GWAS loci. Considering single eQTLs we observed 183 GWAS loci and considering combinatorial eQTLs we observed 129 GWAS loci (Supplementary Figure 13).

Supplementary Figure 13: Singleton and module colocalization with pancreatic traits and disease

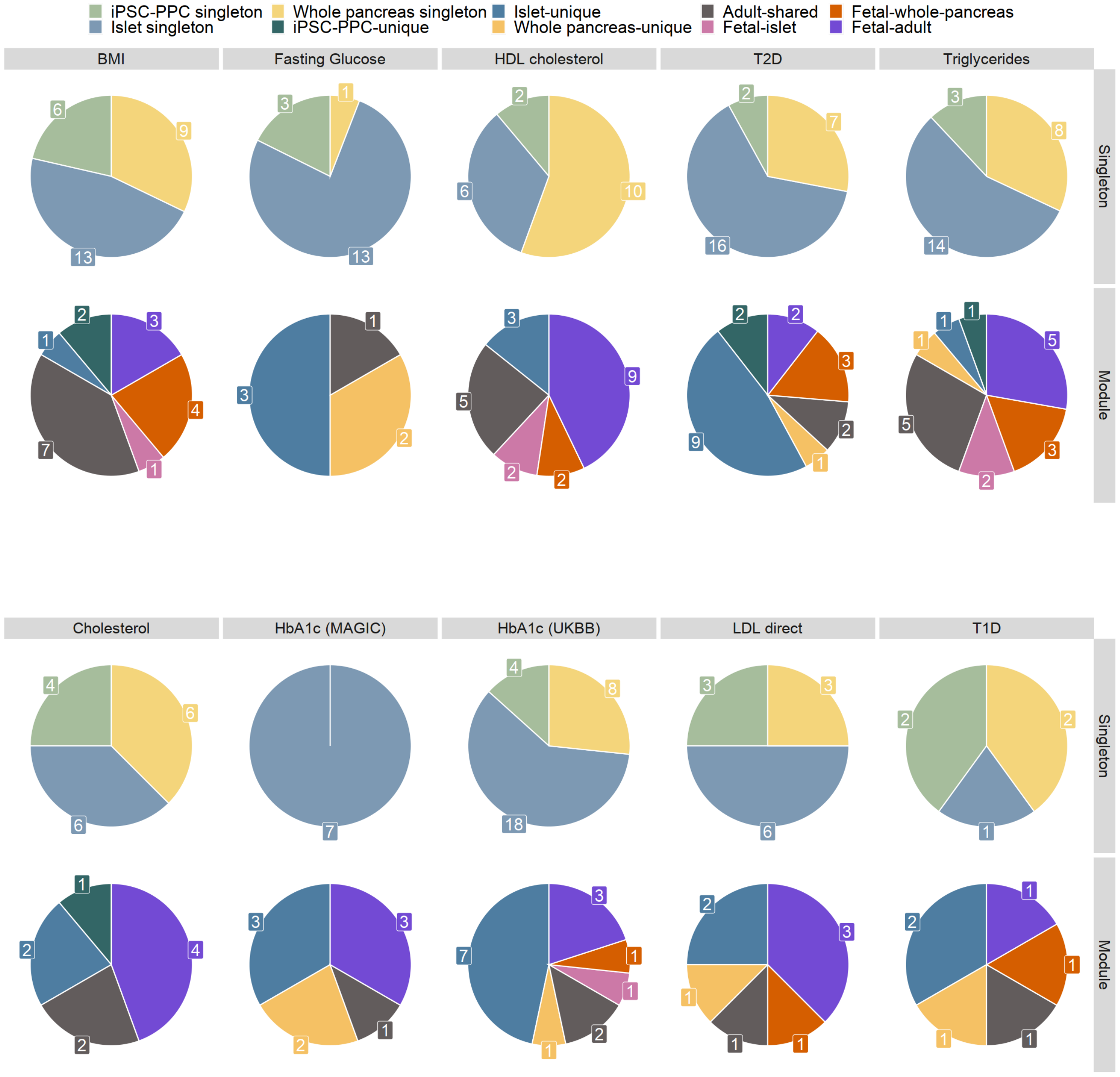

Pie charts summarizing number of colocalizations between eQTLs and GWAS variants for each of the ten traits. Labels indicate the number of singleton eQTLs (top rows) and modules (bottom rows) that colocalized with a GWAS signal. For example, for T1D, two iPSC-PPC-unique singleton (light green), one adult islet-unique singleton (light blue), and two adult whole pancreas-unique singleton (light yellow) eQTLs colocalized with a T1D-risk signal. Similarly, we identified two adult islet-unique eQTL modules (blue), one fetal-adult eQTL modules (orange), and etc., that colocalized with T1D-risk variants.

Supplementary Figure 14: e_gQTL associations for GWAS-associated eGenes in iPSC-PPC

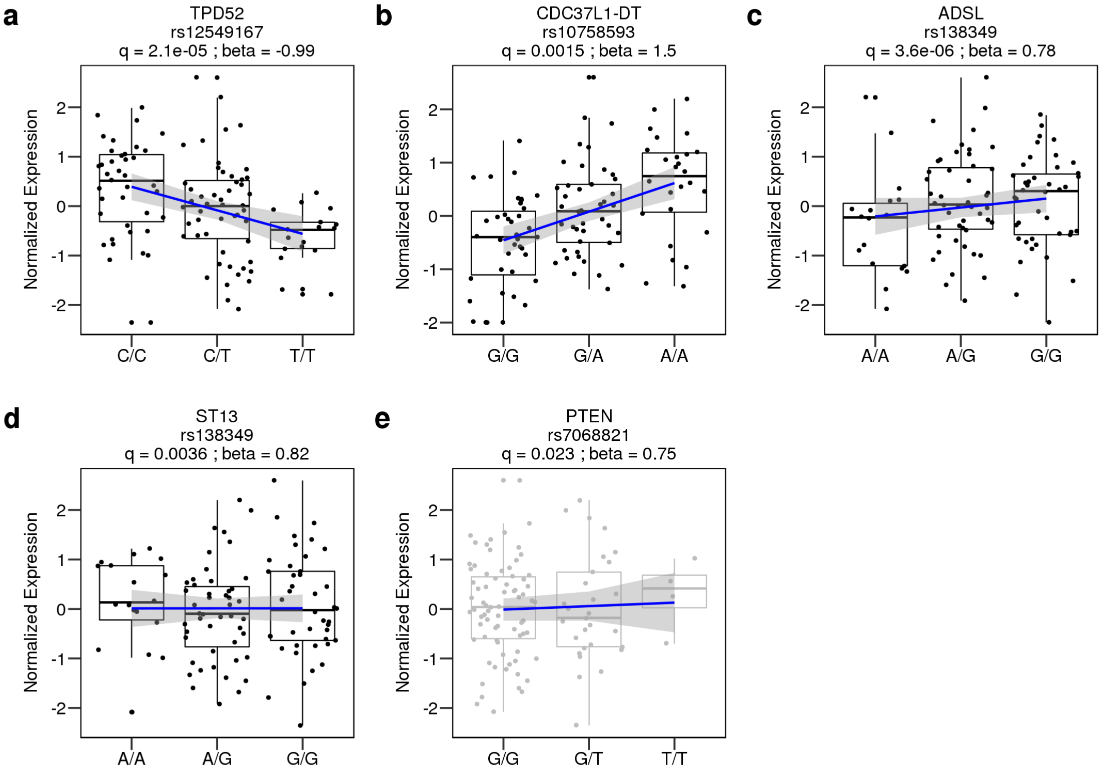

For each candidate susceptibility eGene, we show the association between genotype of the lead candidate causal variant (predicted from GWAS-eQTL colocalization) and their normalized gene expression in iPSC-PPC. Box plots colored in black indicate genes with a significant e_gQTL, while the one colored in gray indicates that it was not associated with genotype. Box plots indicate median interquartile range (IQR), and 1.5 x IQR. Smoothed regression line represents the relationship between genotype and normalized expression (*lm* function in R). We note that *ST13* is an eGene in iPSC-PPC but its e_gQTL signal neither colocalized nor was in LD with *ST13* e_gQTLs in adult islet and adult whole pancreas. The signal was significant (q-value < 0.01) but weak.

Supplementary Figure 15: Pancreatic e_gQTL colocalization with GWAS traits

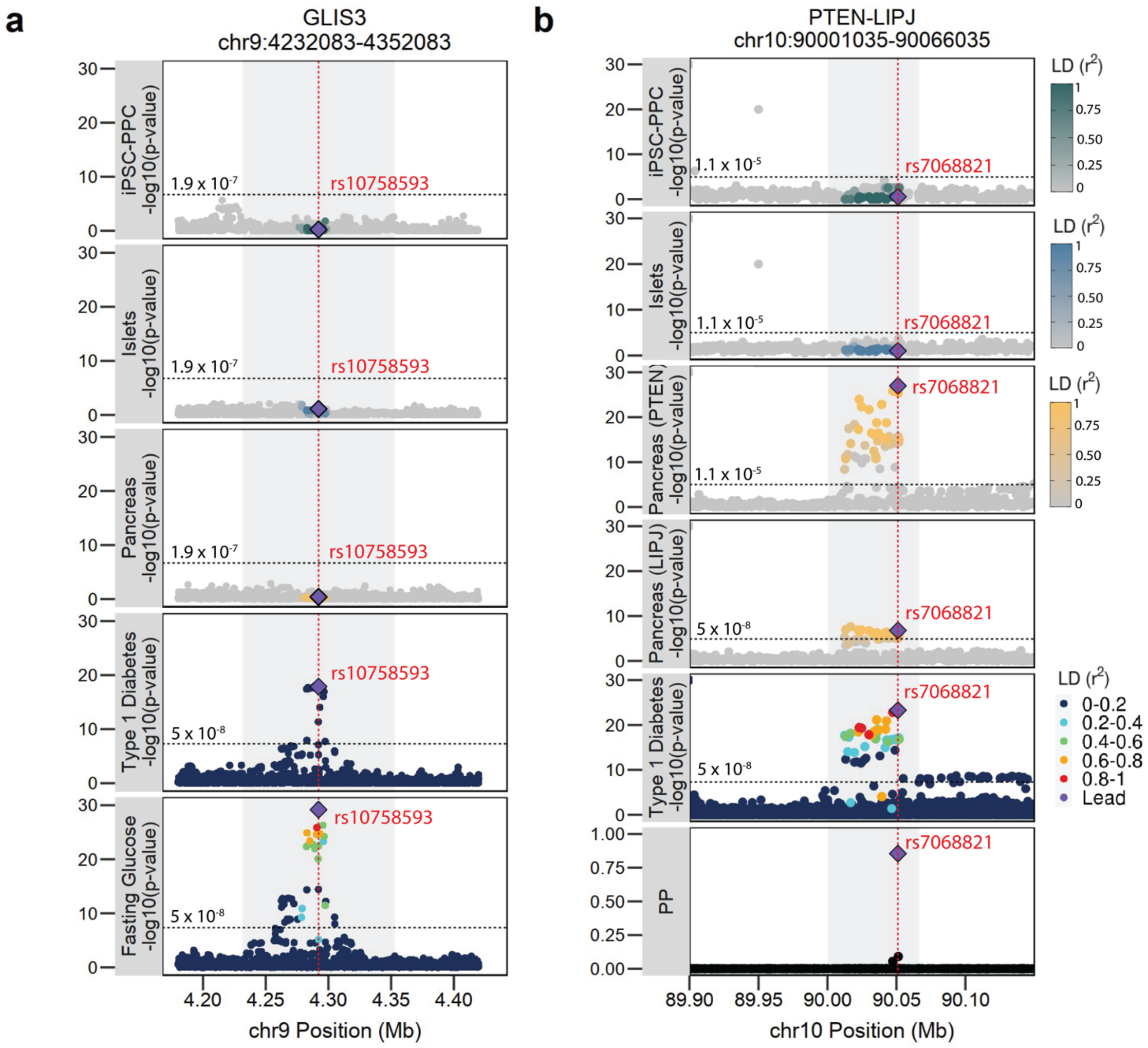

(**a**) This panel shows that the GWAS signals associated with T1D-risk and FG levels in the chr9:423083-4352083 locus did not colocalize with e_gQTLs for *GLIS3* in iPSC-PPC and the adult pancreatic tissues. (**b**) The GWAS signal associated with T1D-risk in the chr10:90001035-90066035 locus colocalized with an adult whole pancreas-unique e_gQTL module containing e_gQTLs for *PTEN* and *LIPJ*. Each point in these plots represents an individual SNP color-coded by LD with the lead candidate causal variant highlighted in purple. The bottom plot in panel **b** shows the posterior probability (PP) of association for each variant being causal for both e_gQTL and GWAS associations. For plotting purposes, we assigned a single p-value for gene-level significance based on Bonferroni-correction (0.05 divided by the number of variants tested for the gene; horizontal line) for the e_gQTL signals. For iPSC-PPC and adult islet in panel **b**, we overlaid eQTL associations for *PTEN, LIPJ*, and nearby genes to show that the locus was not associated with gene expression in the tissues. For GWAS signals, we used p-value = $5x10^{-8}$ to indicate genome-wide significance. Red vertical lines indicate the positions of the lead candidate causal variants underlying GWAS and eQTL colocalization based on maximum PP.

Supplementary Figure 16: e$_{AS}$QTL associations for GWAS-associated eIsoforms in fetal-like iPSC-PPC

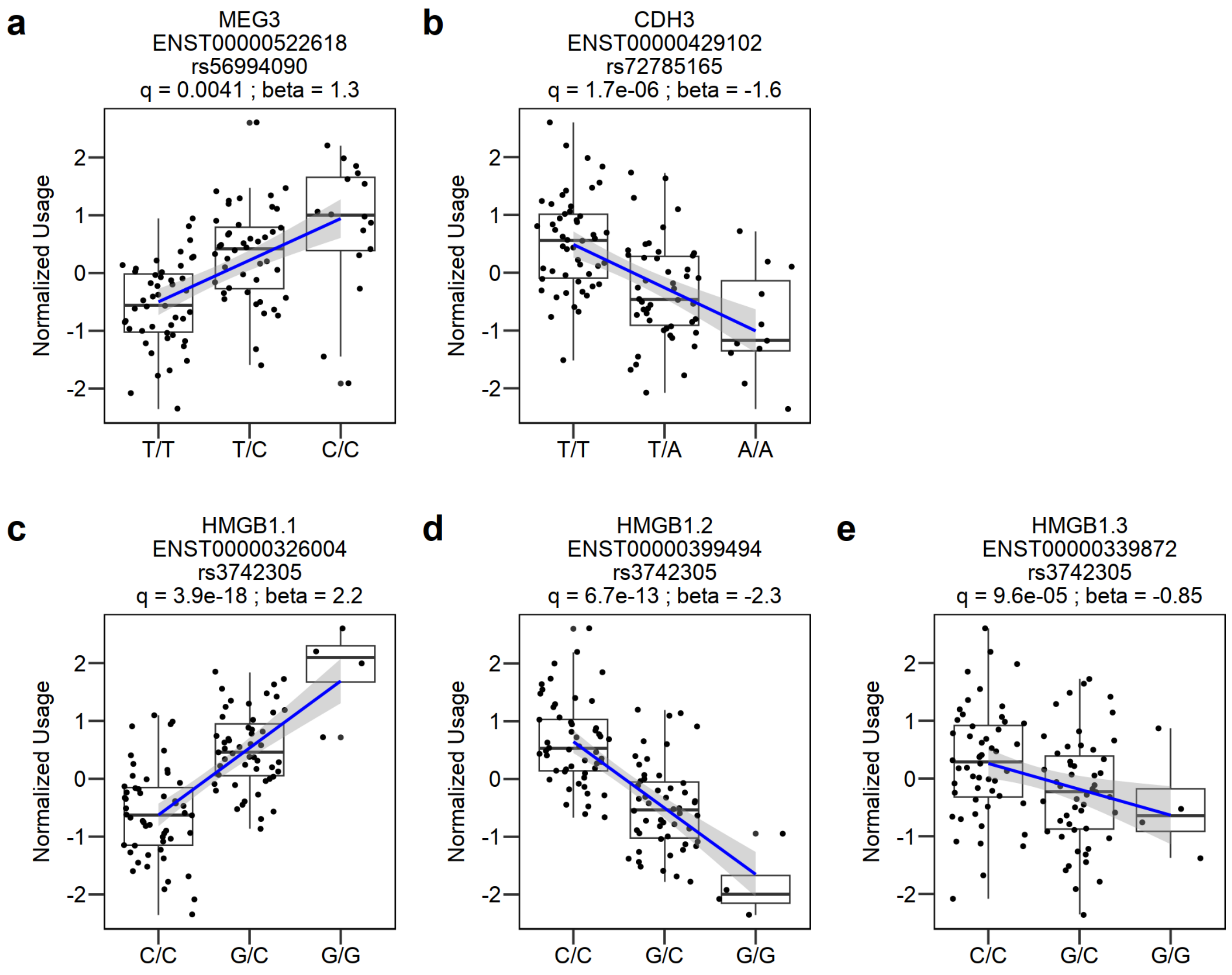

For each candidate susceptibility isoform, we show the association between the genotype of the lead candidate causal variant (predicted from GWAS-eQTL colocalization) and their normalized isoform usage in iPSC-PPC. Box plots indicate median interquartile range (IQR), and 1.5 x IQR. Smoothed regression line indicate the relationship between genotype and normalized expression (*lm* function in R).

Supplementary Figure 17: PEER Factor Optimization

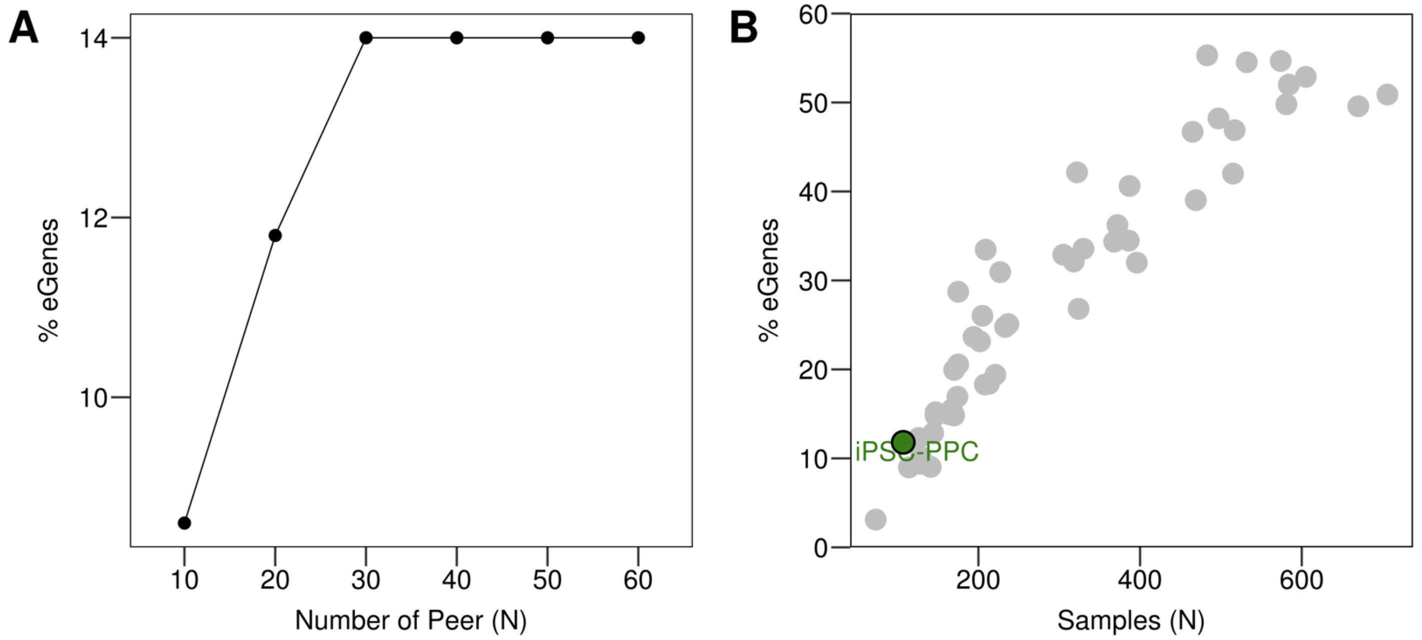

The line plot in panel **a** shows the percentage of eGenes discovered in iPSC-PPC on a random set of 500 genes using PEER factors ranging from 10 to 60 in increments of 10 as covariates. While 30 PEER factors resulted in maximum eGene percentage (14.0%), we selected 20 PEER factors (11.8%) because the percentage of eGenes was comparable to GTEx tissues of similar sample sizes (panel **b**; colored in gray). These results show that compared with GTEx, our study was as well-powered to detect significant eQTL associations.

Supplementary Figure 18: Correlation between eQTL covariates

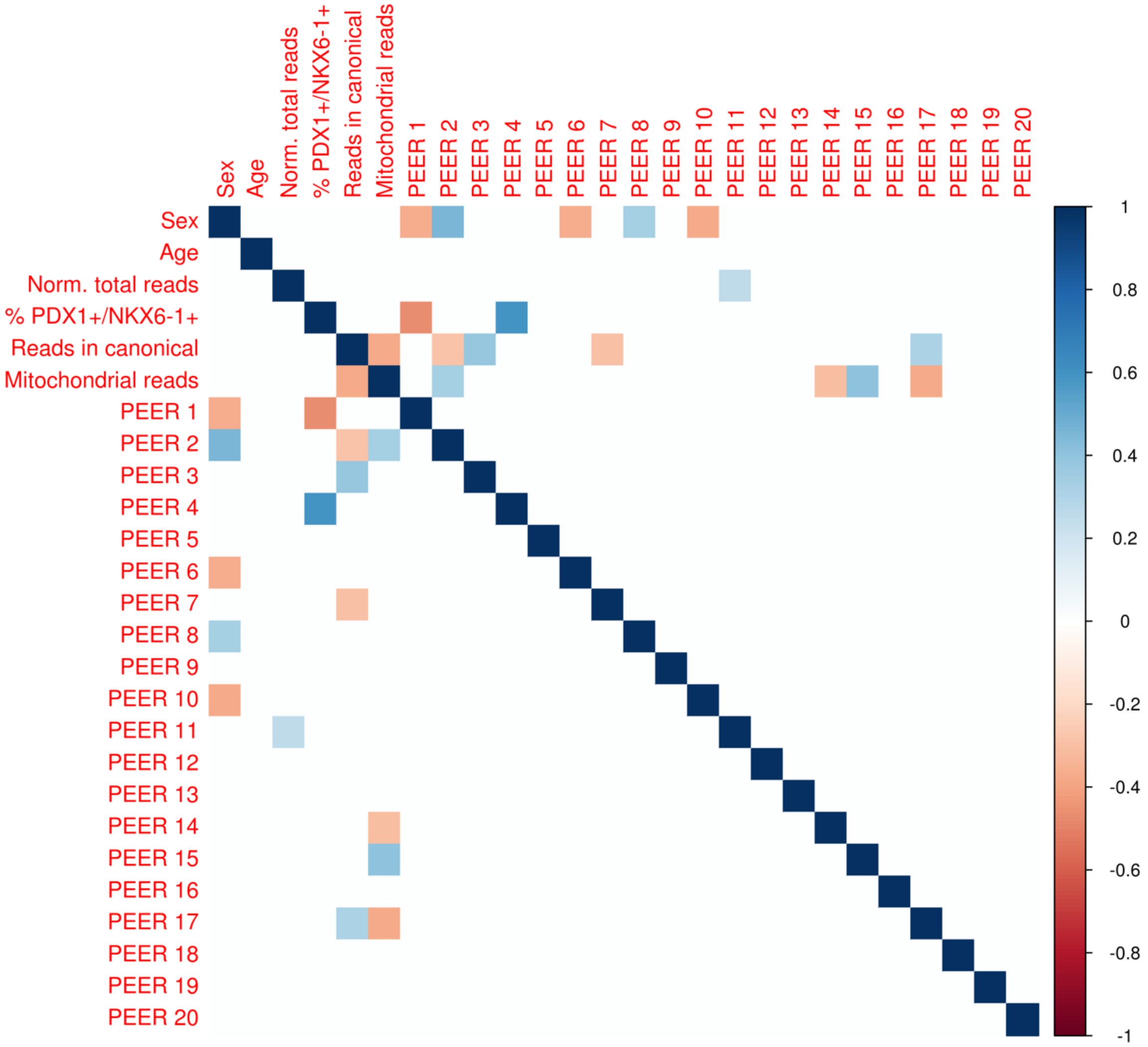

To determine whether PEER factors were correlated with other attributes of iPSC-PPC, we performed a spearman correlation analysis between each pair of covariates used in the linear mixed model for eQTL analysis. We found that PEER factors 1 and 4 were associated with the percentage of cells expressing PDX1^{+}/NKX6-1^{+} based on FACs, therefore accounting for any cellular heterogeneity in iPSC-PPC that could be driving expression variability across the samples.

Supplementary References

1. Veres A, Faust AL, Bushnell HL, et al. Charting cellular identity during human in vitro β-cell differentiation. *Nature*. 2019;569(7756):368-373. doi:10.1038/s41586-019-1168-5

2. D'Antonio M, Nguyen JP, Arthur TD, et al. Fine mapping spatiotemporal mechanisms of genetic variants underlying cardiac traits and disease. *Nat Commun*. 2023;14(1):1132. doi:10.1038/s41467-023-36638-2

3. Garrido-Martín D, Borsari B, Calvo M, Reverter F, Guigó R. Identification and analysis of splicing quantitative trait loci across multiple tissues in the human genome. *Nat Commun*. 2021;12(1):727. doi:10.1038/s41467-020-20578-2

4. The GTEx Consortium. The GTEx Consortium atlas of genetic regulatory effects across human tissues. *Science*. 2020;369(6509):1318-1330. doi:10.1126/science.aaz1776

5. Frankish A, Diekhans M, Ferreira AM, et al. GENCODE reference annotation for the human and mouse genomes. *Nucleic Acids Res*. 2019;47(D1):D766-D773. doi:10.1093/nar/gky955

6. Baralle FE, Giudice J. Alternative splicing as a regulator of development and tissue identity. *Nat Rev Mol Cell Biol*. 2017;18(7):437-451. doi:10.1038/nrm.2017.27

7. Mazin PV, Khaitovich P, Cardoso-Moreira M, Kaessmann H. Alternative splicing during mammalian organ development. *Nat Genet*. 2021;53(6):925-934. doi:10.1038/s41588-021-00851-w

8. Su CH, D D, Tarn WY. Alternative Splicing in Neurogenesis and Brain Development. *Front Mol Biosci*. 2018;5:12. doi:10.3389/fmolb.2018.00012

9. Panopoulos AD, D'Antonio M, Benaglio P, et al. iPSCORE: A Resource of 222 iPSC Lines Enabling Functional Characterization of Genetic Variation across a Variety of Cell Types. *Stem Cell Rep*. 2017;8(4):1086-1100. doi:10.1016/j.stemcr.2017.03.012

10. Newman AM, Steen CB, Liu CL, et al. Determining cell type abundance and expression from bulk tissues with digital cytometry. *Nat Biotechnol*. 2019;37(7):773-782. doi:10.1038/s41587-019-0114-2

11. DeBoever C, Li H, Jakubosky D, et al. Large-Scale Profiling Reveals the Influence of Genetic Variation on Gene Expression in Human Induced Pluripotent Stem Cells. *Cell Stem Cell*. 2017;20(4):533-546.e7. doi:10.1016/j.stem.2017.03.009

12. Fadista J, Vikman P, Laakso EO, et al. Global genomic and transcriptomic analysis of human pancreatic islets reveals novel genes influencing glucose metabolism. *Proc Natl Acad Sci*. 2014;111(38):13924-13929. doi:10.1073/pnas.1402665111

13. GTEx Consortium. Genetic effects on gene expression across human tissues. *Nature*. 2017;550(7675):204-213. doi:10.1038/nature24277

14. Trapnell C, Cacchiarelli D, Grimsby J, et al. The dynamics and regulators of cell fate decisions are revealed by pseudotemporal ordering of single cells. *Nat Biotechnol*. 2014;32(4):381-386. doi:10.1038/nbt.2859

15. Schmidt JV, Matteson PG, Jones BK, Guan XJ, Tilghman SM. The *Dlk1* and *Gtl2* genes are linked and reciprocally imprinted. *Genes Dev*. 2000;14(16):1997-2002. doi:10.1101/gad.14.16.1997

16. Yevtodiyenko A, Schmidt JV. Dlk1 expression marks developing endothelium and sites of branching morphogenesis in the mouse embryo and placenta. *Dev Dyn*. 2006;235(4):1115-1123. doi:10.1002/dvdy.20705

17. Seymour PA. Sox9: A Master Regulator of the Pancreatic Program. *Rev Diabet Stud*. 2014;11(1):51-83. doi:10.1900/RDS.2014.11.51

18. Seymour PA, Freude KK, Tran MN, et al. SOX9 is required for maintenance of the pancreatic progenitor cell pool. *Proc Natl Acad Sci*. 2007;104(6):1865-1870. doi:10.1073/pnas.0609217104

19. Aigha II, Abdelalim EM. NKX6.1 transcription factor: a crucial regulator of pancreatic β cell development, identity, and proliferation. *Stem Cell Res Ther*. 2020;11(1):459. doi:10.1186/s13287-020-01977-0

20. Van Hoof D, D'Amour KA, German MS. Derivation of insulin-producing cells from human embryonic stem cells. *Stem Cell Res*. 2009;3(2-3):73-87. doi:10.1016/j.scr.2009.08.003

21. Oliver-Krasinski JM, Stoffers DA. On the origin of the β cell. *Genes Dev*. 2008;22(15):1998-2021. doi:10.1101/gad.1670808
