# Supplementary material for: eQTL mapping in fetal-like pancreatic progenitor cells reveals early developmental insights into diabetes risk
Source: Nat Commun. 2023 Oct 30;14:6928. doi: 10.1038/s41467-023-42560-4 (PMC10616100; doi:10.1038/s41467-023-42560-4)
Supplement: Supplementary file 3 — Description of Additional Supplementary Files [file 41467_2023_42560_MOESM3_ESM.pdf]

Description of Additional Supplementary Files

Supplementary Data 1: Subject Information

The first sheet contains information about each of the 106 iPSCORE individuals included in this study. Columns are: **ipscore_id**: subject iPSCORE ID, **subject_uuid**: universally unique identifier (UUID) for the subject, **sex**: sex, **age**: age at the time of enrollment, **most_similar_1kg_pop**: most similar 1000 Genomes Phase 3 superpopulation from our previous study [35], **family_uuid**: family UUID, **wgs_uuid**: whole-genome sequencing (WGS) sample UUID, and **pc1-20**: the first 20 genotype principal components (PC) accounting for global ancestry. The second sheet contains the kinship matrix used for eQTL mapping, where the row and column names correspond to the WGS UUID's.

Supplementary Data 2: Sample Information

This table contains information about the 107 iPSC-PPC samples used in this study. Columns are: **udid**: unique differentiation ID, **subject_uuid**: subject UUID, **wgs_uuid**: WGS sample UUID, **live_scrna_uuid**: sample UUIDs for scRNA-seq samples prepared with fresh cells, **cryo_scrna_pool_uuid**: sample UUIDs for scRNA-seq samples prepared after cryopreservation of the cells (cells from four iPSC-PPC samples were pooled into one sample and cells from another three iPSC-PPC samples were pooled into a second sample), **cryo_scrna_pool_name**: name labels for the pooled scRNA-seq samples, **live_scrna_pi_hat**: PI_HAT indicating sample match to the subject (from plink genome), **bulk_rna_uuid**: sample UUIDs for each bulk RNA-seq sample, **ipsc_clone**: clone of the iPSC line used for differentiation, **ipsc_passage_at_monolayer**: passage of the iPSC line at monolayer, **day15_pdx1_nkx6.1**: percentage of cells expressing PDX1 and NKX6-1 at day 15 of differentiation measured by flow cytometry, **day15_pdx1**: percentage of cells expressing PDX1 at day 15 of differentiation measured by flow cytometry, **day15_nkx6.1**: percentage of cells expressing NKX6-1 at day 15 of differentiation measured by flow cytometry, **total_reads**: total reads sequenced (note: divide this number by 2 to get the number of paired reads), **total_reads_norm**: normalized total number of reads sequenced, **uniquely_mapped_reads_canonical_chromosomes**: percentage of uniquely mapped reads in autosomal and sex chromosomes, **pct_intergenic_bases**: percentage of bases that mapped to intergenic regions of genomic DNA (from Picard RnaSeqMetrics), **pct_mrna_bases**: percentage of bases that mapped to regions corresponding to UTRs and coding regions of mRNA transcripts (from Picard RnaSeqMetrics), **pct_duplicates**: percentage of duplicate reads (from samtools flagstat), **pct_mitochondrial_reads**: percentage of reads mapping to mitochondrial chromosome (from samtools idxstats), **bulk_rna_pi_hat**: PI_HAT indicating sample match to the subject (from plink genome), **peer1-20**: the 20 PEER factors used in eQTL mapping in iPSC-PPC.

Supplementary Data 3: scRNA-seq metadata

For each of the 84,225 single cells that passed quality control, we provide: **cell_id**: ID of the single cell, **barcode:** cell barcode, **cryo_scrna_pool_name:** name labels for the pooled scRNA-seq samples (only for samples prepared using

cryopreserved cells), **sample_preparation**: method of scRNA-seq preparation ("fresh" indicates that the scRNA-seq sample was prepared using fresh cells immediately after differentiation, "Cryopreserved" indicates that the scRNA-seq sample was prepared using cryopreserved cells), **udid**: unique differentiation ID, **scrna_uuid**: UUID for the scRNA-seq sample the cells came from (corresponds to **live_scrna_uuid** and **cryo_scrna_pool_uuid** columns in Supplementary Data 2), **wgs_uuid**: WGS sample UUID, **subject_uuid**: subject UUID, **ncount_rna**: total number of molecules detected within a cell, **nfeature_rna**: total number of genes detected in each cell, **percent_mt**: proportion of transcripts mapping to mitochondrial genes, **cluster_res.0.05**: cluster ID numbers for each cell at resolution 0.05, **cluster_res.0.08**: cluster ID numbers for each cell at resolution 0.08, **cluster_res.0.1**: cluster ID numbers for each cell at resolution 0.1, **celltype**: cell type labels for each cell at resolution 0.08, **UMAP_1** and **UMAP_2**: UMAP coordinates of each cell. The WGS UUIDs were mapped to each single cell using Demuxlet [97] and then mapped to the subject UUID. A Seurat R Object for filtered and integrated dataset is available on Figshare: https://doi.org/10.6084/m9.figshare.21836208.

Supplementary Data 4: Differentially expressed genes in scRNA-seq clusters

For each celltype and gene, we report: **pct.1**: percentage of cells that expressed the gene in the cell type cluster, **pct.2**: percentage of cells that expressed the gene outside of the celltype cluster, **avg_log2FC:** the log fold-change of the average expression between the two groups, **p_val**: p-value from two-sided Wilcox Rank-Sum test, **p_val_adj**: adjusted p-value based on Bonferroni correction using all features in the dataset. Genes with adjusted p-value ≤ 0.05 were considered differentially expressed.

Supplementary Data 5: Cellular deconvolution of iPSC-PPC bulk RNA-seq

This table reports the estimated relative proportions of each of the eight cell types (clusters) identified in the scRNA-seq data for each of the 107 iPSC-PPC bulk RNA-seq samples (Supplementary Figure 4). Columns are: **UDID**: unique differentiation ID, **Early_DE**: the estimated relative proportion of early DE cells, **Early_Ductal**: the estimated relative proportion of early ductal cells, **Early_PPC**: the estimated relative proportion of early PPC cells, **Endocrine**: the estimated relative proportion of endocrine cells, **iPSC**: the estimate relative proportion of iPSC cells, **Late_PPC**: the estimated relative proportion of late PPC cells, **Mesendoderm**: the estimated relative proportion of mesendoderm cells, **Rep_Late_PPC**: the estimated relative proportion of replicating late PPC cells. The relative proportions were estimated by CIBERSORTx [108] deconvolution (Supplementary Figure 7B). In sheet 2, we provide the cell type signature matrix used for the deconvolution.

Supplementary Data 6: PCA and pseudotime analyses on iPSC, iPSC-PPC, and adult pancreatic tissues

We report results from PCA and pseudotime analysis on 107 iPSC-PPCs, 213 iPSCs [37], 87 adult islets [38], and 176 adult whole pancreas samples (phs000424.v7.p2) (Supplementary Figure 9). For each sample, we report: **sample_id**: bulk RNA-seq ID, **tissue**: tissue type, **pseudotime**: pseudotime inferred by Monocle [107] (see Methods), **pc1-10:** principal components for each sample after principal components analysis on the top variable genes (see Methods). Sample IDs were assigned as

the following: sample bulk RNA-seq UUIDs for iPSCORE samples (iPSC and iPSC-PPC), sample accession IDs from GEO for adult islet samples (GSE50398), and SRA accession IDs for adult whole pancreas samples (phs000424.v7).

Supplementary Data 7: Lead variants for all e_gQTLs and e_iQTLs in iPSC-PPC

The table reports the lead eSNP for each eQTL discovered in iPSC-PPC. We provide: **eqtl_phenotype**: the phenotype the eQTL was associated with (gene expression or isoform usage), **transcript_id**: transcript ID, **gene_id:** gene ID, **gene_name**: gene name, **discovery_order**: discovery order of the eQTL, where 0 represents primary eQTL and 1-4 represents conditional eQTLs, **eqtl_id**: eQTL ID assigned as [tissue_type]_[discovery_order]_[transcript_ID], **snp**: lead variant ID assigned as [chromosome]_[position]_[reference allele]_[alternate allele], **chrom**: the lead variant's chromosome, **pos:** hg19 position of the lead variant, **ref**: reference allele of the lead variant, **alt**: alternate allele of the lead variant, **beta**: the lead variant's effect size on gene expression or isoform usage, **se**: standard error, **pval**: eQTL p-value for the association between genotype of the lead variant and gene expression or isoform usage, **tests**: number of independent variants used for eigenMT [109] p-value correction, **fdr**: FDR-corrected eQTL p-value calculated by eigenMT, **qval**: q-value from Benjamini-Hochberg correction of fdr, **egene:** TRUE/FALSE indicating whether the eQTL is significant or not with q-value threshold < 0.01. Full summary statistics are available on Figshare as a tar zipped directory containing text files for each gene and isoform tested: https://doi.org/10.6084/m9.figshare.21899496 and https://doi.org/10.6084/m9.figshare.21899499.

Supplementary Data 8: Correlation between eQTL effect sizes and TF binding affinity

This table contains results for the association analysis between eQTLs and TF binding affinity. We report: **snp.pp_threshold:** threshold used for individual variant causal posterior probability, **eqtl_phenotype**: the molecular phenotype tested for association with genetic variation (gene expression or isoform usage), **cor**: estimated measure of association from Pearson's product-moment correlation, **pval**: p-value of the correlation test.

Supplementary Data 9: Colocalization Results between iPSC-PPC and Adult eQTLs (PP $\geq$ 80%)

Sheet 1: Colocalization between iPSC-PPC egQTL and eiQTLs. The table reports colocalization results for the 410 eGenes with H3 and/or H4 associations between their e_gQTLs and corresponding e_iQTLs.

Sheet 2: eGene colocalization between iPSC-PPC and adult islet. The table reports colocalization results for the 795 shared eGenes with H3 and/or H4 association between iPSC-PPC and adult islet e_gQTLs.

Sheet 3: Input for generating e_gQTL networks. The table reports colocalization results for all 7,893 e_gQTL pairs between the three pancreatic tissues.

Sheet 4: Input for generating e_{AS}QTL networks. The table reports colocalization results for all 4,868 e_{AS}QTLs pairs between the three pancreatic tissues.

In each table, we provide: **eqtl_id.1**: eQTL ID for one of the two eQTLs being colocalized, **eqtl_id.2**: eQTL ID for thw second eQTL being colocalized, **transcript_id.1**: transcript ID for eqtl_id.1, **transcript_id.2**: transcript ID for eqtl_id.2, **gene_id.1**:

gene ID for eqtl_id.1, **gene_id.2**: gene ID for eqtl_id.2, **gene_name.1**: gene name for eqtl_id.1, **gene_name.2**: gene name for eqtl_id.2, **eqtl_phenotype.1**: the molecular phenotype tested for association with genetic variation (gene expression or isoform usage) for **eqtl_id.1**, **eqtl_phenotype.2**: the molecular phenotype tested for association with genetic variation (gene expression or isoform usage) for **eqtl_id.2**, **tissue.1**: tissue the first eQTL was detected in, **tissue.2**: the tissue the second eQTL was detected in, **discovery_order**: discovery order of the eQTL, where 0 represents primary eQTL and 1-4 represents conditional eQTLs, **nsnps**: number of variants used to test for colocalization (obtained from *coloc.abf*), **PP.H0.abf**: posterior probability of H0 model (no causal variant), **PP.H1.abf**: posterior probability of H1 model (causal variant for trait 1 only), **PP.H2.abf**: posterior probability of H2 model (causal variant for trait 2 only), **PP.H3.abf**: posterior probability of H3 model (two distinct causal variants), **PP.H4.abf**: posterior probability of H4 model (one common causal variant), **likely_model**: model with the strongest evidence of being true based on highest posterior probability, **max_model_pp**: the maximum PP across the models, **topsnp**: the lead predicted causal variant if PP.H4.abf was true, **topsnp_pp**: the posterior probability that **topsnp** is causal for the association with the molecular phenotype. Multiple variants may be listed as the lead predicted causal variants if they share the same maximal posterior probability. eQTL IDs were assigned as [tissue_type]_[discovery_order]_[transcript_ID].

Supplementary Data 10: eQTL Annotation for each iPSC-PPC and Adult eQTLs

The table describes the annotations for each e_gQTL and e_{AS}QTL in the three pancreatic tissues. We provide: **eqtl_id:** eQTL ID assigned as [tissue_type]_[discovery_order]_[transcript_ID], **transcript_id:** transcript ID, **gene_id**: gene ID, **gene_name**: gene_name, **tissue**: tissue source the eQTL was detected in, **eqtl_phenotype**: the molecular phenotype tested for association with genetic variation (gene expression or alternative splicing), **eqtl_type**: label indicating whether the eQTL was a singleton or combinatorial, **module_id**: module ID assigned as [eQTL_phenotype]_[chromosome]_[number], where "GE" represents eQTL modules associated withe gene expression and "AS" represents eQTL modules associated with alternative splicing (module IDs are given to only combinatorial eQTLs, see also Supplementary Data 13), **expressed_ipsc_ppc**: TRUE/FALSE indicating whether the gene was expressed and tested for genetic association in iPSC, **expressed_islet**: TRUE/FALSE indicating whether the gene was expressed and tested for genetic association in adult islets, **expressed_pancreas**: TRUE/FALSE indicating whether the gene was expressed and tested for genetic association in adult whole pancreas, **LD_ipsc_ppc:** TRUE/FALSE indicating whether the eQTL was in LD with nearby iPSC-PPC eQTLs, **LD_islet**: TRUE/FALSE indicating whether the eQTL was in LD with nearby adult islet eQTLs, **LD_pancreas**: TRUE/FALSE indicating whether the eQTL was in LD with nearby adult whole pancreas eQTLs, **islet_egene_overlap**: labels describing the eGene overlap between iPSC-PPC and adult islet eQTLs in the module (zero means there were no adult islet eQTLs in the module, same means that all eGenes overlapped between iPSC-PPC and adult islet eQTLs, partial means that there was at least one shared eGene and at least one different eGene between iPSC-PPC and adult islet eQTLs, and different means that there was no overlap in eGenes between iPSC-PPC and adult islet eQTLs), **pancreas_egene_overlap**: labels describing the eGene overlap between iPSC-PPC and adult whole pancreas eQTLs in the module (zero means there were no adult whole pancreas eQTLs in the module, same means that all eGenes were the same between all iPSC-PPC and adult whole pancreas

eQTLs, partial means that there was at least one shared eGene and at least one different eGene between iPSC-PPC and adult whole pancreas eQTLs, and different means that there was no overlap in eGenes between iPSC-PPC and adult whole pancreas eQTLs), **module_pass**: TRUE/FALSE indicating whether the module passed threshold requirements (see Methods), **category_annotation**: labels for for each eQTL based on whether it was unique to a single tissue, shared with another tissue, or was a singleton or combinatorial (see below or Methods for descriptions for each category), **notes**: comments describing why the eQTL was annotated as "ambiguous" or "module_failed". Below, we describe what each category annotation means in the table. Descriptions are also provided in the Methods.

1) "ipsc_ppc singleton": the eQTL was an iPSC-PPC-unique singleton eQTL
2) "islet singleton": the eQTL was an adult islet-unique singleton eQTL
3) "whole-pancreas singleton": the eQTL was an adult whole pancreas-unique singleton eQTL
4) "ipsc_ppc-unique": the eQTL was in an iPSC-PPC-unique module
5) "islet-unique": the eQTL was in an adult islet-unique module
6) "whole-pancreas-unique": the eQTL was in an adult whole pancreas-unique module
7) "adult-shared": the eQTL was in an adult-shared module (shared between adult islets and adult whole pancreas; module contained at least one eQTL from adult islets, at least one eQTL from adult whole pancreas, and zero eQTLs from iPSC-PPC)
8) "fetal-islet": the eQTL was in a fetal-islet module (shared between iPSC-PPC and adult islets; module contained at least one eQTL from iPSC-PPC, at least one eQTL from adult islets, and zero eQTLs from adult whole pancreas)
9) "fetal-whole-pancreas": the eQTL was in a fetal-whole-pancreas module (shared between iPSC-PPC and adult whole pancreas; module contained at least one eQTL from iPSC-PPC, at least one eQTL from adult whole pancreas, and zero eQTLs from adult islets)
10) "fetal-adult": the eQTL was in a fetal-adult module (shared between iPSC-PPC and the two adult tissues; module contained at least one eQTL from iPSC-PPC, at least one eQTL from adult islets, and at least one eQTL from adult whole pancreas)
11) "module_failed": the eQTL was excluded due to being in a module that did not satisfy threshold requirements (see Methods)
12) "ambiguous": the eQTL was excluded due to being in LD with a nearby eQTL. If the eQTL was in a module, the eQTL was in LD with an eQTL in a different tissue. If the eQTL was a singleton eQTL, the eQTL was in LD with another nearby eQTL in the same or different tissue. Tissue-specificity for this eQTL could not be determined and therefore excluded from downstream analyses.

Supplementary Data 11: Network Modules of iPSC-PPC and Adult eQTLs

The table provides information for each e_gQTL (sheet 1) and $e_{AS}QTL$ (sheet 2) module. Specifically, we provide: **module_id**: module ID assigned as [eQTL_phenotype]_[chromosome]_[number], where "GE" represents eQTL modules associated withe gene expression and "AS" represents eQTL modules associated with alternative splicing (module IDs are given to

only combinatorial eQTLs), **associations:** all eQTL associations in the module by their eQTL IDs, **number_assocs:** number of eQTL associations in the module, **number_ipsc_ppc_assocs**: number of iPSC-PPC eQTL associations in the module, **number_islet_assocs**: number of adult islet eQTL associations in the module, **number_pancreas_assocs:** number of adult whole pancreas eQTL assocations in the module, **islet_egene_overlap**: labels describing the eGene overlap between iPSC-PPC and adult islet eQTLs in the module (zero means there were no adult islet eQTLs in the module, same means that all eGenes overlapped between iPSC-PPC and adult islet eQTLs, partial means that there was at least one shared eGene and at least one different eGene between iPSC-PPC and adult islet eQTLs, and different means that there was no overlap in eGenes between iPSC-PPC and adult islet eQTLs), **pancreas_egene_overlap**: labels describing the eGene overlap between iPSC-PPC and adult whole pancreas eQTLs in the module (zero means there were no adult whole pancreas eQTLs in the module, same means that all eGenes were the same between all iPSC-PPC and adult whole pancreas eQTLs, partial means that there was at least one shared eGene and at least one different eGene between iPSC-PPC and adult whole pancreas eQTLs, and different means that there was no overlap in eGenes between iPSC-PPC and adult whole pancreas eQTLs), **egene_overlap_category**: eGene overlap category shown in Figure 4A and Supplementary Figure 11C, **module_pass**: TRUE/FALSE indicating whether the module passed threshold requirements (see Methods), **category_annotation**: labels for for each eQTL based on whether it was unique to a single tissue, shared with another tissue, or was a singleton or combinatorial (see below or Methods for descriptions for each category), **notes**: comments describing why the eQTL was annotated as "ambiguous" or "module_failed". Below, we describe what each category annotation means in this table. Descriptions are also provided in the Methods.

1) "ipsc_ppc-unique": the module contained only iPSC-PPC eQTLs
2) "islet-unique": the module contained only adult islet eQTLs
3) "whole-pancreas-unique": the module contained only adult whole pancreas eQTLs
4) "adult-shared": the module was an adult-shared module and contained only eQTLs from adult islet and adult whole pancreas
5) "fetal-islet": the module contained only iPSC-PPC and adult islet eQTLs
6) "fetal-whole-pancreas": the module contained only iPSC_PPC and adult whole pancreas eQTLs
7) "fetal-adult": the module contained iPSC-PPC, adult islet, and adult whole pancreas eQTLs
8) "module_failed": the module did not pass threshold requirements (see Methods)
9) "ambiguous": the module contained an eQTL that is in LD with another eQTL in a different tissue

Supplementary Data 12: Chromatin State Enrichments of iPSC-PPC-unique eQTL Singletons and Modules

The table reports chromatin enrichment results for iPSC-PPC-unique singleton and combinatorial e_gQTLs at various SNP.PP thresholds. We provide: **snp.pp_threshold:** threshold used for individual variant causal posterior probability, **category_annotation**: labels for for each eQTL based on whether it was unique to a single tissue, shared with another tissue, or was a singleton or combinatorial (see above or Methods for descriptions for each category), **eqtl_type**: label indicating whether the eQTL was a singleton or combinatorial, **chromatin_annotation**: chromatin state annotation, **tissue**:

the tissue where the chromatin annotations were drived from, **study**: the study source for the chromosome annotation, **estimate**: odds-ratio, **pval**: p-value of significance for two-sided Fisher's Exact Test comparing the fraction of variants overlapping the chromotation annotations against a null set of variants, **qval:** Benjamini-Hochberg correction of the p-value. **ci1** and **ci2**: lower and upper limits of the confidence interval.

Supplementary Data 13: Colocalization of iPSC-PPC and Adult eQTLs with Pancreatic GWAS Traits

The table reports information about the 312 GWAS loci that colocalized with iPSC-PPC and/or adult eQTLs with PP.H4 ≥ 80%. Note that a GWAS locus can be listed more than once if it colocalized with more than one eQTL (only observed for modules). In this table, we provide: **gwas_locus_id**: eQTL-GWAS locus ID assigned as [eQTL module ID or eQTL singleton ID] [trait_id], **eqtl_id**: eQTL ID assigned as [tissue_type]_[discovery_order]_[transcript_ID], **transcript_id**: transcript ID**,** **gene_id**: gene ID, **gene_name**: gene name, **eqtl_phenotype**: the molecular phenotype tested for association with genetic variation (gene expression or alternative splicing), **tissue**: tissue source the eQTL was detected in, **eqtl_type**: label indicating whether the eQTL was a singleton or combinatorial, **module_id**: module ID assigned as [eQTL_phenotype]_[chromosome]_[number], where "GE" represents eQTL modules associated withe gene expression and "AS" represents eQTL modules associated with alternative splicing (module IDs are given to only combinatorial eQTLs, see also Supplementary Data 13), **trait_id**: ID for the GWAS trait, **description**: description of the GWAS trait, **study_source**: study source of the GWAS summary statistics, **nsnps**: number of variants used to test for colocalization between GWAS and eQTL (obtained from *coloc.abf*), **PP.H0.abf**: posterior probability of H0 model (no causal variant), **PP.H1.abf**: posterior probability of H1 model (causal variant for trait 1 only), **PP.H2.abf**: posterior probability of H2 model (causal variant for trait 2 only), **PP.H3.abf**: posterior probability of H3 model (two distinct causal variants), **PP.H4.abf**: posterior probability of H4 model (one common causal variant), **max_model_pp**: the maximum PP across the models, **likely_model**: model with the strongest evidence of being true based on highest posterior probability, **topsnp**: the lead predicted causal variant if PP.H4.abf was true, **topsnp_pp**: the posterior probability that **topsnp** is causal for the association with the molecular phenotype, **topsnp_gwas_pval**: GWAS p-value for the association between the GWAS trait and the variant, **topsnp_eqtl_pval**: eQTL p-value for the association between gene expression/alternative splicing and the variant, **islet_egene_overlap**: labels describing the eGene overlap between iPSC-PPC and adult islet eQTLs in the module (zero means there were no adult islet eQTLs in the module, same means that all eGenes overlapped between iPSC-PPC and adult islet eQTLs, partial means that there was at least one shared eGene and at least one different eGene between iPSC-PPC and adult islet eQTLs, and different means that there was no overlap in eGenes between iPSC-PPC and adult islet eQTLs), **pancreas_egene_overlap**: labels describing the eGene overlap between iPSC-PPC and adult whole pancreas eQTLs in the module (zero means there were no adult whole pancreas eQTLs in the module, same means that all eGenes were the same between all iPSC-PPC and adult whole pancreas eQTLs, partial means that there was at least one shared eGene and at least one different eGene between iPSC-PPC and adult whole pancreas eQTLs, and different means that there was no overlap in eGenes between iPSC-PPC and adult whole pancreas eQTLs), **category_annotation**: labels for for each eQTL based on whether it was unique to a single tissue, shared with another tissue, or was a singleton or combinatorial (see

Supplementary Data 12 and 13 for the description of each category), **used_to_finemap**: the eQTL used to finemap the GWAS signal (relevant for eQTL modules that had multiple eQTL signals that colocalized with the GWAS signal), **cs_size**: number of putative causal variants in the 99% credible set. Multiple variants may be listed as the lead predicted causal variants (**topsnp**) if they share the same maximal posterior probability

Supplementary Data 14: 99% Credible Sets for iPSC-PPC eQTLs Associated with GWAS

This table contains the 99% credible sets for each of the 312 GWAS loci described in Supplementary Data 15. The eQTLs used to construct the 99% credible sets can be found in Supplementary Data 15 with the **used_to_finemap** column. In this table, we provide: **gwas_locus_id**: eQTL-GWAS locus ID assigned as [eQTL module ID or eQTL singleton ID] [trait_id], **trait_id**: ID for the GWAS trait, **description**: description of the GWAS trait, **study_source**: study source of the GWAS summary statistics, **eqtl_id**: eQTL ID assigned as [tissue_type]_[discovery_order]_[transcript_ID], **snp**: ID for the variant in the credible set, colocalization statistics from *coloc.abf* (columns F-T), **SNP.PP.H4**: posterior probability for the variant being causal for eQTL and GWAS associations from *coloc.abf*.

Supplementary Data 15: LD analysis with non-pancreatic GTEx tissues

This table contains results for the LD analysis conducted between the 16 iPSC-PPC-unique e_gQTLs that colocalized with GWAS signals and the e_gQTLs in the 48 non-pancreatic tissues in the GTEx dataset version 8 [10]. We provide: **eqtl_id:** eQTL ID assigned as [tissue_type]_[discovery_order]_[transcript_ID], **eqtl_phenotype**: the molecular phenotype tested for association with genetic variation (gene expression or alternative splicing), **eqtl_type**: label indicating whether the eQTL was a singleton or combinatorial, **tissue**: tissue source the eQTL was detected in, **transcript_id**: transcript ID, **gene_id**: gene ID, **gene_name**: gene name, **category_annotation**: labels for for each eQTL based on whether it was unique to a single tissue, shared with another tissue, or was a singleton or combinatorial (see Supplementary Data 12 and 13 for the description of each category), **in_ld_other_gtex**: TRUE/FALSE indicating whether the e_gQTL is in LD with an adult adult e_gQTL
