# Supplementary material for: eQTL mapping in fetal-like pancreatic progenitor cells reveals early developmental insights into diabetes risk
Source: Nat Commun. 2023 Oct 30;14:6928. doi: 10.1038/s41467-023-42560-4 (PMC10616100; doi:10.1038/s41467-023-42560-4)
Supplement: Supplementary file 19 — Reporting Summary [file 41467_2023_42560_MOESM19_ESM.pdf]

nature portfolio

Corresponding author(s): Kelly A. Frazer

Last updated by author(s): Jul 27, 2023

Reporting Summary

Nature Portfolio wishes to improve the reproducibility of the work that we publish. This form provides structure for consistency and transparency in reporting. For further information on Nature Portfolio policies, see our Editorial Policies and the Editorial Policy Checklist.

Statistics

For all statistical analyses, confirm that the following items are present in the figure legend, table legend, main text, or Methods section.

n/a	Confirmed	
☐	☒	The exact sample size (*n*) for each experimental group/condition, given as a discrete number and unit of measurement
☐	☒	A statement on whether measurements were taken from distinct samples or whether the same sample was measured repeatedly
☐	☒	The statistical test(s) used AND whether they are one- or two-sided<br>*Only common tests should be described solely by name; describe more complex techniques in the Methods section.*
☐	☒	A description of all covariates tested
☐	☒	A description of any assumptions or corrections, such as tests of normality and adjustment for multiple comparisons
☐	☒	A full description of the statistical parameters including central tendency (e.g. means) or other basic estimates (e.g. regression coefficient) AND variation (e.g. standard deviation) or associated estimates of uncertainty (e.g. confidence intervals)
☐	☒	For null hypothesis testing, the test statistic (e.g. *F*, *t*, *r*) with confidence intervals, effect sizes, degrees of freedom and *P* value noted<br>*Give P values as exact values whenever suitable.*
☐	☒	For Bayesian analysis, information on the choice of priors and Markov chain Monte Carlo settings
☐	☒	For hierarchical and complex designs, identification of the appropriate level for tests and full reporting of outcomes
☐	☒	Estimates of effect sizes (e.g. Cohen's *d*, Pearson's *r*), indicating how they were calculated

Our web collection on statistics for biologists contains articles on many of the points above.

Software and code

Policy information about availability of computer code

Data collection	We used the following tools: R V.4.2.1; STAR V.2.7.3a; Gencode V.34lift37; sambamba V.0.6.7; biobambam2 V.2.0.95; RSEM V.1.2.20; CIBERSORTx website; bcftools V.1.9; limix V.3.0.4; plink 1.90b3x; bedtools V.2.27.1; numpy V.1.20.3; pandas V.1.3.5; samtools V.1.9<br>We used the following R packages: preprocessCore 1.58.0; coloc 5.1.0.1; peer 1.0; igraph 1.3.4
Data analysis	Scripts developed to perform this study are available in: https://github.com/jenniferngp/iPSC_PPC_eQTL_Project

For manuscripts utilizing custom algorithms or software that are central to the research but not yet described in published literature, software must be made available to editors and reviewers. We strongly encourage code deposition in a community repository (e.g. GitHub). See the Nature Portfolio guidelines for submitting code & software for further information.

Data

Policy information about availability of data

All manuscripts must include a data availability statement. This statement should provide the following information, where applicable:

- Accession codes, unique identifiers, or web links for publicly available datasets
- A description of any restrictions on data availability
- For clinical datasets or third party data, please ensure that the statement adheres to our policy

The iPSC-PPC scRNA-seq and bulk RNA-seq data generated in this study have been deposited in the GEO database under accession codes GSE152610 and GSE182758, respectively. The WGS data used in this study for iPSCORE individuals were obtained as a VCF file from phs001325.v3. The reference gene annotation

file for aligning bulk RNA-seq data of iPSC-PPC were obtained from GENCODE release version 34 in GRCh37 as a GTF file (https://www.gencodegenes.org/human/release_34.html). The bulk RNA-seq data for iPSC, adult islet, and adult whole pancreas samples used in PCA and pseudotime analyses were obtained from phs000924, GSE50398, and phs000424, respectively. eQTL summary statistics for adult whole pancreas and islet samples were obtained from the GTEx Data Repository (https://console.cloud.google.com/storage/browser/gtex-resources) and a previously published study 11 (https://zenodo.org/record/3408356), respectively. GWAS summary statistics were obtained from the Pan UK BioBank resource (https://pan.ukbb.broadinstitute.org/), the MAGIC (Meta-Analyses of Glucose and Insulin-related traits) Consortium (https://magicinvestigators.org/downloads/; https://doi.org/10.1038/s41588-021-00852-9), the DIAMANTE Consortium (https://diagram-consortium.org/downloads.html; http://doi.org/10.1038/s41588-018-0241-6), and a previously published study 3. Full summary statistics for all eQTLs, fine-mapping, and supporting data for figures and supplemental tables have been deposited in Figshare: https://figshare.com/projects/Large-scale_eQTL_analysis_of_iPSC-PPC/156987.

Research involving human participants, their data, or biological material

Policy information about studies with human participants or human data. See also policy information about sex, gender (identity/presentation), and sexual orientation and race, ethnicity and racism.

Reporting on sex and gender	NA
Reporting on race, ethnicity, or other socially relevant groupings	NA
Population characteristics	NA
Recruitment	NA
Ethics oversight	NA

Note that full information on the approval of the study protocol must also be provided in the manuscript.

Field-specific reporting

Please select the one below that is the best fit for your research. If you are not sure, read the appropriate sections before making your selection.

☒ Life sciences ☐ Behavioural & social sciences ☐ Ecological, evolutionary & environmental sciences

For a reference copy of the document with all sections, see nature.com/documents/nr-reporting-summary-flat.pdf

Life sciences study design

All studies must disclose on these points even when the disclosure is negative.

Sample size	Our sample size for conducting iPSC-derived pancreatic progenitor eQTL analysis is 107, making it the largest eQTL study (as we currently know) on fetal developmental pancreatic cells.
Data exclusions	No data was excluded.
Replication	Replication was not performed because it is not a standard procedure in eQTL studies.
Randomization	Randomization was not performed because it is not a standard procedure in eQTL studies. Covariates such as sex, sequencing quality and global ancestry were included in the linear mixed models developed in this study.
Blinding	Blinding was not performed because it is not a standard procedure in eQTL studies.

Reporting for specific materials, systems and methods

We require information from authors about some types of materials, experimental systems and methods used in many studies. Here, indicate whether each material, system or method listed is relevant to your study. If you are not sure if a list item applies to your research, read the appropriate section before selecting a response.

Materials & experimental systems

n/a	Involved in the study
☐	☒ Antibodies
☒	☐ Eukaryotic cell lines
☒	☐ Palaeontology and archaeology
☒	☐ Animals and other organisms
☒	☐ Clinical data
☒	☐ Dual use research of concern
☒	☐ Plants

Methods

n/a	Involved in the study
☒	☐ ChIP-seq
☐	☒ Flow cytometry
☒	☐ MRI-based neuroimaging

Antibodies

Antibodies used	PE Mouse anti-PDX1 Clone-658A5 (BD Biosciences; 1:10), Alexa Fluor 647 Mouse anti-NKX6.1 Clone R11-560 (BD Bioscience; 1:10), PE Mouse anti-IgG1 κ R-PE Clone MOPC-21 (BD Biosciences), Alexa Fluor 647 Mouse anti IgG1 κ Isotype Clone MOPC-21 (BD Biosciences).
Validation	Both PE Mouse anti-PDX1 Clone-658A5 (BD Biosciences) and Alexa Fluor 647 Mouse anti-NKX6.1 Clone R11-560 (BD Bioscience) were validated by the manufacturer; they were found to be reactive with both mouse and human PDX-1 and NKX6-1, respectively. We also used the appropriate class control in our experiments: PE Mouse anti-IgG1 κ R-PE Clone MOPC-21 (BD Biosciences) and Alexa Fluor 647 Mouse anti IgG1 κ Isotype Clone MOPC-21 (BD Biosciences).

Flow Cytometry

Plots

Confirm that:

☒ The axis labels state the marker and fluorochrome used (e.g. CD4-FITC).

☒ The axis scales are clearly visible. Include numbers along axes only for bottom left plot of group (a 'group' is an analysis of identical markers).

☒ All plots are contour plots with outliers or pseudocolor plots.

☒ A numerical value for number of cells or percentage (with statistics) is provided.

Methodology

Sample preparation	At least 2 x 10^6 cells were fixed and permeabilized using the Fixation/Permeabilized Solution Kit with BD GolgiStop TM (BD Biosciences) following the manufacturer's recommendations. Cells were resuspended in 1x BD Perm/Wash TM Buffer at a concentration of 1 x 10^7 cells/ml. For each flow cytometry staining, 2.5 x 10^5 cells were stained for 75 minutes at room temperature with PE Mouse anti-PDX1 Clone-658A5 (BD Biosciences; 1:10) and Alexa Fluor 647 Mouse anti-NKX6.1 Clone R11-560 (BD Bioscience; 1:10), or with the appropriate class control antibodies: PE Mouse anti-IgG1 κ R-PE Clone MOPC-21 (BD Biosciences) and Alexa Fluor 647 Mouse anti IgG1 κ Isotype Clone MOPC-21 (BD Biosciences). Stained cells were washed three times, resuspended in PBS containing 1% BSA and 1% formaldehyde, and immediately analyzed using FACS Canto II flow cytometer (BD Biosciences).
Instrument	FACS Canto II flow cytometer (BD Biosciences)
Software	FlowJo software version 10.4
Cell population abundance	Double-positive PDX1+ and NKX6-1+ cells ranged from 9.4% to 93.1% (median: 74%) across the 107 iPSC-derived pancreatic progenitor samples. Cell population abundance was accounted for in the eQTL analysis.
Gating strategy	Single cells were selected based on the Forward scatter and Side scatter and all debris, and double cells were excluded. Next, for each flow cytometry staining experiment, cells were stained with individual appropriate class control antibodies: PE Mouse anti-IgG1 κ R-PE Clone MOPC-21 (BD Biosciences) and Alexa Fluor 647 Mouse anti IgG1 κ Isotype Clone MOPC-21 (BD Biosciences) to set up the PDX1 and NKX6.1 double positive cells quadrant gating.

☒ Tick this box to confirm that a figure exemplifying the gating strategy is provided in the Supplementary Information.
